# The tumor suppressor LKB1 regulates starvation-induced autophagy under systemic metabolic stress

**DOI:** 10.1038/s41598-017-07116-9

**Published:** 2017-08-04

**Authors:** Laurie A. Mans, Laia Querol Cano, Jason van Pelt, Panagiota Giardoglou, Willem-Jan Keune, Anna-Pavlina G. Haramis

**Affiliations:** 1 Institute of Biology, Leiden University, Sylviusweg 72, 2333 BE Leiden, The Netherlands; 2 Department of Biochemistry, Netherlands Cancer Institute, Plesmanlaan 121, 1066 CX Amsterdam, The Netherlands; 3 Radboud Institute for Molecular Life Sciences - Tumour immunology department, Geert Grooteplein 28, 6525 GA Nijmegen, The Netherlands

## Abstract

Autophagy is an evolutionarily conserved process that degrades cellular components to restore energy homeostasis under limited nutrient conditions. How this starvation-induced autophagy is regulated at the whole-body level is not fully understood. Here, we show that the tumor suppressor Lkb1, which activates the key energy sensor AMPK, also regulates starvation-induced autophagy at the organismal level. Lkb1-deficient zebrafish larvae fail to activate autophagy in response to nutrient restriction upon yolk termination, shown by reduced levels of the autophagy-activating proteins Atg5, Lc3-II and Becn1, and aberrant accumulation of the cargo receptor and autophagy substrate p62. We demonstrate that the autophagy defect in *lkb1* mutants can be partially rescued by inhibiting mTOR signaling but not by inhibiting the PI3K pathway. Interestingly, mTOR-independent activation of autophagy restores degradation of the aberrantly accumulated p62 in *lkb1* mutants and prolongs their survival. Our data uncover a novel critical role for Lkb1 in regulating starvation-induced autophagy at the organismal level, providing mechanistic insight into metabolic adaptation during development.

## Introduction

Autophagy is a highly conserved, multi-step intracellular process of self-degradation. Under basal conditions, autophagy eliminates damaged proteins and organelles from cells, serving a housekeeping/recycling function. However, upon metabolic stress, starvation–induced autophagy serves to provide substrates for biosynthesis and energy production in order to maintain cellular homeostasis^1, 2^. The importance of autophagy for cellular and organismal health is showcased by the fact that defects in autophagy have been linked to neurodegeneration, cancer, aging and metabolic syndrome^1^.

Starvation-induced autophagy promotes survival in the *Drosophila* fat body^3^ and in *Caenorhabditis elegans* ^4^. A critical role for autophagy in surviving the metabolic stress at birth has also been demonstrated in mammals: mice deficient in Atg5 (autophagy protein 5, an E3 ubiquitin ligase necessary for autophagosomal elongation) survive foetal development, but die within one day after birth, exhibiting severe hypoglycaemia and hypolipidaemia^2^. However, the regulation of systemic starvation-induced autophagy is not well understood.

Autophagy-induction in response to energetic stress is triggered by the activation of AMPK-activated protein kinase (AMPK)^5, 6^, a key, evolutionarily conserved energy sensor. AMPK activation restores energy homeostasis at the cellular and organismal levels^7^ by many different pathways, including via inhibition of the mechanistic target of rapamycin (mTOR)^8^, a conserved serine-threonine kinase involved in nutrient sensing, growth and proliferation^9, 10^. AMPK itself is activated by multiple mechanisms including phosphorylation by the tumor suppressor LKB1/STK11 kinase in response to increased AMP or ADP levels in the cell^11, 12^. Because of the role of AMPK as a central energy checkpoint in the cell, these findings link LKB1 signaling to energy metabolism control, positioning LKB1 as a critical mediator of the effects of low energy on cell viability^11, 13^. Accordingly, cells lacking LKB1 undergo apoptosis under metabolic stress as they are unable to respond to energy deficiency and restore homeostasis^11^. LKB1/AMPK signaling is also important for long-term survival under nutrient-limiting conditions during *C. elegans* dauer (diapause) stage^14^. However, the early embryonic lethality of both *Lkb1* mutant^15^ and *Ampka1/a2* double mutant mice^16^ has precluded analysis of the *in vivo* role of LKB1/AMPK in physiological processes occurring at later developmental stages in vertebrates, such as during metabolic stress at birth.

The LKB1/AMPK axis is a negative regulator of mTOR signaling^8^ and mTOR signaling is a known inhibitor of autophagy^17^. However, LKB1 also activates 12 other AMPK-related kinases^18^, and many mTOR-dependent and mTOR-independent autophagy regulators exist^19^.

LKB1/AMPK regulation of mTOR has been linked to the regulation of autophagy in different settings, for example in autophagy stimulated by fluid flow over the primary cilium of epithelial cells^20^, and in cancer cells^21^. Furthermore, AMPK directly stimulates autophagy via the ULK1/Atg1 phosphorylation^22, 23^. And LKB1 may also stimulate autophagy by stabilizing p27, thereby linking nutrient sensing to cell-cycle progression^13^. However, whether and how LKB1 signaling regulates systemic starvation-induced autophagy in vertebrates is currently unknown.

The regulation of systemic metabolism and autophagy are often studied in zebrafish because of its small size and vertebrate physiology^24, 25^. Importantly, fundamental principles of energy homeostasis are highly conserved between humans and zebrafish^26, 27^. Autophagy has critical functions during zebrafish embryonic development^28^, with autophagy-defective animals displaying abnormal heart development^29^, which is also seen in mice^30^. Zebrafish are also a valuable model for studying tissue regeneration, and autophagy has been shown to be required for the regeneration of amputated caudal fins^31^. Like mammals, zebrafish also experience metabolic stress at birth, when the maternal nutrient supply (yolk) is depleted. The metabolic stress at birth in mammals is accompanied by induction of gluconeogenesis^32, 33^ and of autophagy^2, 34^. While induction of gluconeogenesis also serves as a mechanism to restore energy homeostasis in zebrafish^35^, a role for autophagy during this metabolic transition has not been investigated.

We previously used TILLING^36^ to generate zebrafish mutants that carried a point mutation leading to a stop codon in the kinase domain of Lkb1 *(stk11* ^*hu1968*^). These mutants survived gastrulation and early embryonic development but died prematurely from starvation at 7 to 8 days post-fertilization (dpf). Our experiments, impossible to conduct in mice due to the early embryonic lethality of *Lkb1* knock-out mice^15^, established Lkb1 as a critical regulator of whole-body energy homeostasis^37^. Zebrafish *lkb1 (stk11* ^*hu1968*^) mutants are unable to cope with the energetic stress induced upon yolk depletion and fail to adapt their metabolism to lower nutrient levels. *lkb1* mutants are indistinguishable from wild type (wt) siblings while their maternal nutrient supply is still present, until 5–6 dpf. However, they die within 1–2 days following yolk depletion whereas wt larvae can survive without food until 13–14 dpf^38^.

The zebrafish *lkb1* mutant phenotype is reminiscent of the *Atg5* knock-out mice that appear normal until birth but die soon after, due to their inability to cope with the metabolic stress at birth^2^. This resemblance prompted us to investigate the autophagy status in the *lkb1* mutants to study the role of Lkb1 in regulation of systemic starvation-induced autophagy.

We show that Lkb1-deficient larvae fail to activate autophagy in response to nutrient restriction. Furthermore, we demonstrate aberrant accumulation of the autophagy adaptor and substrate p62 in *lkb1* mutants, confirming impaired autophagy. Genetic or chemical induction of autophagy in *lkb1* mutants prolongs their survival, while suppression of autophagy shortens it. Survival prolongation only occurs when degradation of p62 is restored. We therefore show that autophagy is essential to survive the feeding-fasting transition in zebrafish, and identify Lkb1 as a critical regulator of whole-body starvation-induced autophagy in vertebrates.

## Results

### *lkb1* mutants fail to activate autophagy under nutrient limitation

To determine if autophagy initiation and maintenance is affected in the *lkb1* mutants we previously generated^37^, we analyzed wt and *lkb1* larvae between 5–7 dpf during the metabolic transition following yolk depletion. *lkb1* mutants are indistinguishable from wt larvae up to day 5–6 dpf^37^ (while there is still yolk). We chose this time window also because the morphological *lkb1* phenotype of flattened intestine and darkened liver is apparent at 7 dpf^37^ and the majority of *lkb1* mutants die at 8 dpf (Supplementary Fig. S1). Autophagic activity is commonly monitored by accumulation of the membrane-bound form of MAP1LC3B (microtubule-associated proteins 1 A/1B light chain 3B, Atg8 in yeast; Lc3B in zebrafish), Lc3-II, which is a ubiquitin-like protein^39^ that localizes in autophagosomal membranes upon induction of autophagy. To enable visualization of Lc3-II accumulation in autophagosomes, we first blocked the fusion of autophagosomes with lysosomes by treating the larvae with 2.5 μM chloroquine^40^ for 14 hours (h) before analysis.

**Figure 1:**
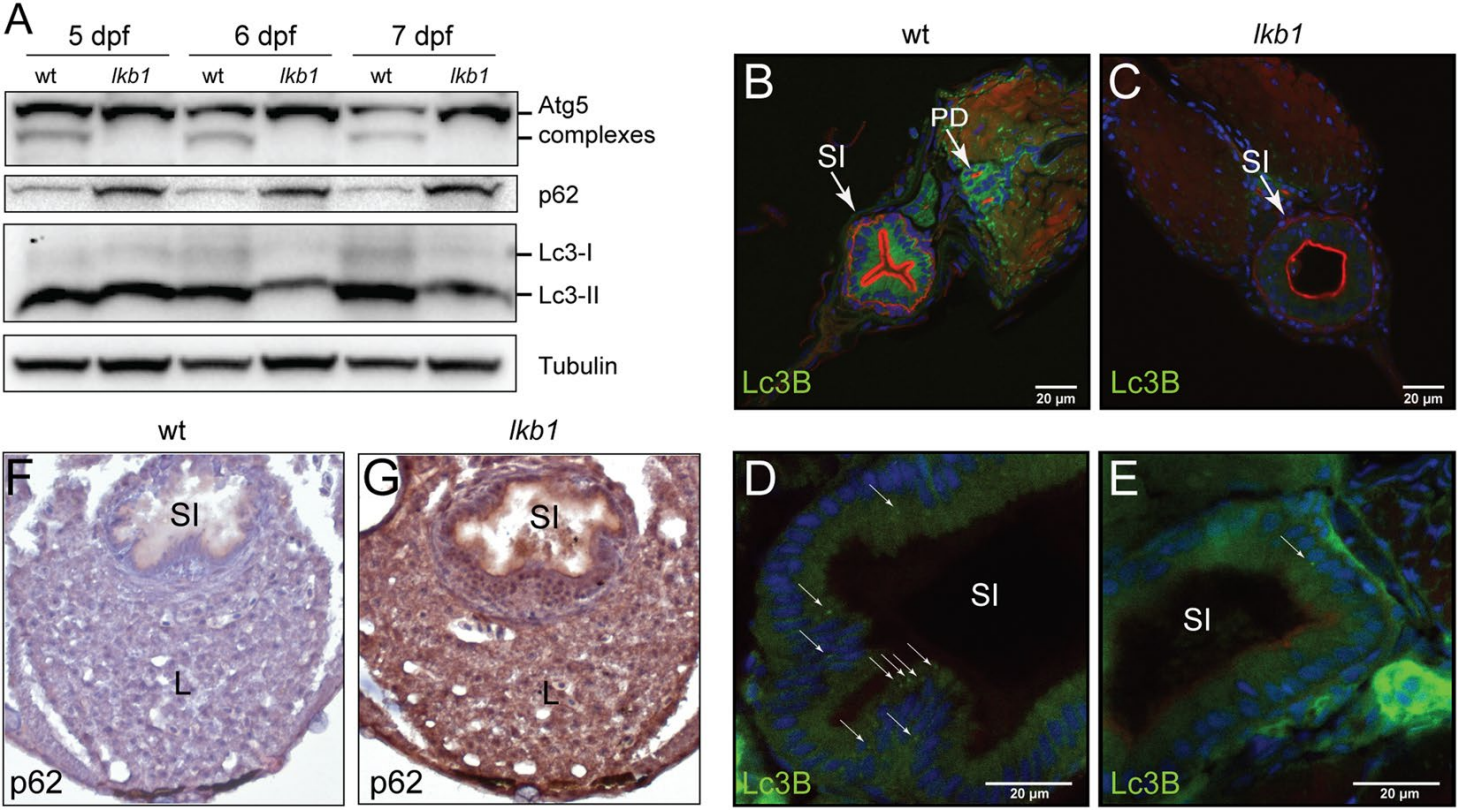
*lkb1* mutant larvae show impaired activation of autophagy following yolk depletion. (**A**) Representative Western blot analysis of Lc3-II, Atg5, p62 and Tubulin (loading control) in total protein lysates of wt and *lkb1* trunks between 5–7 dpf. Larvae were treated with chloroquine (2.5 μM) for 14 h prior to processing. The marked decrease in Lc3-II and Atg5-containing complexes together with the p62 accumulation indicate impaired autophagy in *lkb1* larvae. Uncropped images of the blots are shown in Supplementary Fig. S7A–C. (**B**–**E**) Transverse vibratome sections (150 μm) of intestine of 7 dpf wt and *lkb1* mutants stained with anti-LC3B antibody (green), rhodamine-phalloidin to detect F-actin (red) and DAPI to detect nuclei (blue). Lc3B staining in the *lkb1* intestine is barely detectable (**C**,**E**) and more foci of intense staining were visible in wt sections compared to sections from *lkb1* mutants. (**F**,**G**) Immunohistochemical analysis of transverse paraffin sections (5 μm) of liver and intestine of 7 dpf wt and *lkb1* larvae reveals high levels of p62 accumulation in *lkb1* liver and intestine. Magnification: 40×. PD: pronephric ducts; L: liver; SI: intestine.

We found that Lc3-II protein levels were lower in *lkb1* mutant larvae compared to their wt siblings after yolk depletion at 6 and 7 dpf (Fig. 1A). Note that the Lc3 antibody in zebrafish recognizes predominantly the cleaved Lc3B-II form, which still accurately reflects autophagic activity^41^ (Supplementary Fig. S2A), and we were only able to detect a faint signal for Lc3B-I in zebrafish lysates (Fig. 1A). To verify this result, we used an alternative marker of autophagy by analyzing the expression of Atg5-containing protein complexes during development. We found that while the expression of the common ~56 KD complex was unaffected, the ~47 KD complex, which is indicative of autophagy induction^31^ was undetectable in the *lkb1* mutants (Fig. 1A). Finally, we also assessed the levels of Beclin 1 (Becn1), a protein involved in autophagosome nucleation^30, 42^ and also commonly used as an autophagy indicator. Becn1 expression was also strongly reduced in the *lkb1* mutants (Supplementary Fig. S2B). While Lc3-II and Becn1 levels progressively increased in wt larvae between 5–7 dpf, indicating upregulation of autophagy upon yolk termination, *lkb1* mutants did not show such an increase, suggesting they fail to activate autophagy under nutrient limiting conditions (Fig. 1A, and Supplementary Fig. S2B).

To examine the spatial distribution of autophagy, we performed immunofluorescence analysis with antibodies against Lc3B on transverse liver and intestine sections of *lkb1* and wt larvae at 7 dpf. Lc3B expression was strongly reduced in *lkb1* intestines and livers as compared to their wt counterparts (Fig. 1B–E and Supplementary Fig. S2E,F), confirming and supporting the immunoblotting results. Furthermore, immunohistochemistry (IHC) against Becn1 on transverse sections of wt and *lkb1* mutants showed markedly reduced Becn1 staining in the *lkb1* mutants compared to their wt counterparts (Supplementary Fig. S2C,D).

To further confirm that activation of autophagy is impaired in *lkb1* mutants, we monitored expression levels of the p62 protein (also known as sequestosome 1, (SQSTM1). p62 is an adaptor protein that targets ubiquitinated proteins or organelles that bind to it for selective autophagy^43^. Accumulation of p62 has been observed in mouse AMPK-deficient fibroblasts^44^, and is associated with liver toxicity in autophagy-deficient mouse liver^45^. p62 itself is also an autophagy substrate, thus accumulation of p62 levels is a marker for impaired autophagy^46^. In agreement with our model that *lkb1* mutants have impaired autophagy, Western blot analysis of p62 levels at 5, 6 and 7 dpf showed progressive accumulation of p62 specifically in the *lkb1* mutants (Fig. 1A). IHC performed on transverse sections of *lkb1* intestine and liver confirmed a marked accumulation of p62 in *lkb1* larvae, whereas wt siblings were devoid of staining (Fig. 1F,G).

Collectively, these findings demonstrate that whole-body autophagy is impaired in *lkb1* mutants during the feeding-fasting transition in zebrafish, which could contribute to their premature death.

### Abrogation of autophagy further decreases survival of *lkb1* mutants

**Figure 2:**
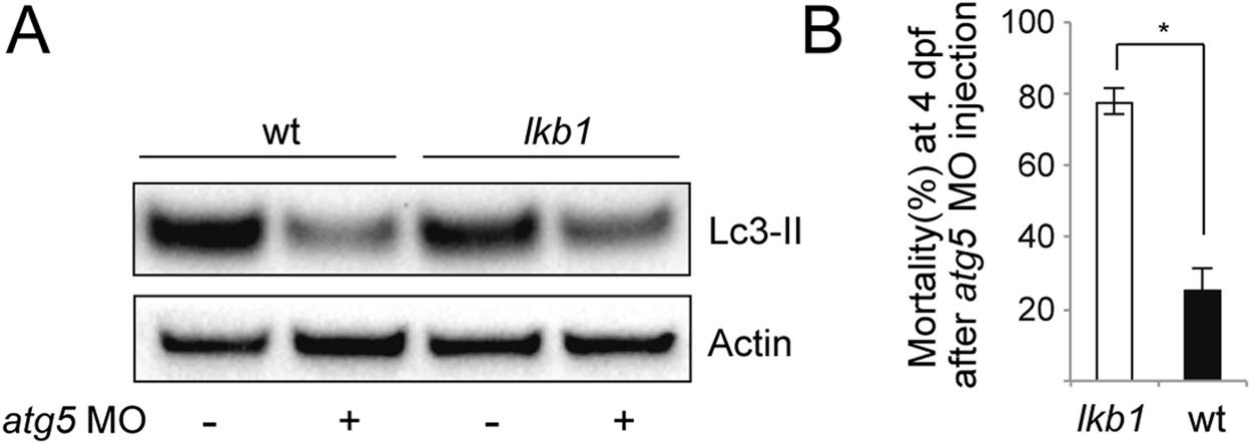
Inhibition of autophagy shortens survival of *lkb1* larvae. (**A**) Representative Western blot analysis of Lc3-II and Actin (loading control) in total protein lysates of trunks of surviving wt and *lkb1* larvae at 4 dpf that were injected with an *atg5*MO at the one-cell stage, and controls. Larvae were previously treated with 2.5 μM chloroquine for 14 h. Atg5 knock-down led to downregulation of Lc3-II levels in both wt and *lkb1* mutants. Uncropped images of the blots are shown in Supplementary Fig. S8. (**B**) Graph depicting mortality percentages of *atg5*MO-injected wt and *lkb1* larvae at 4 dpf. Data represent the means ± standard errors of the means (SEM) and are pooled from two independent experiments (n = 80/experiment). *P value < 0.05.

To investigate the effect of inhibiting autophagy on *lkb1* larvae survival prior to yolk depletion, we blocked autophagosome formation using an antisense morpholino oligonucleotide (MO), that targets the translational start-site of *atg5* mRNA^28, 47^, *atg5*MO. We confirmed that injection of *atg5*MO abolishes Atg5 protein expression (Supplementary Fig. S3). Atg5-knockdown led to a reduction in Lc3-II levels compared to the negative control in both wt and *lkb1* mutants at 4 dpf, before yolk depletion, confirming autophagy suppression upon *atg5*MO injection (Fig. 2A). While all un-injected wt and *lkb1* larvae were alive at 4 dpf, a significant number of *atg5*MO-injected embryos were found dead at that time point. Genotyping all larvae at 4 dpf revealed that 75% of *atg5*MO-injected *lkb1* larvae had died compared to only 25% of *atg5*MO-injected wt larvae (Fig. 2B). Thus, *lkb1* mutants, which fail to induce autophagy at the metabolic transition, are also more sensitive to autophagy inhibition at earlier embryonic stages.

### The autophagy defect in *lkb1* mutants can be ameliorated by mTOR-dependent and -independent mechanisms

**Figure 3:**
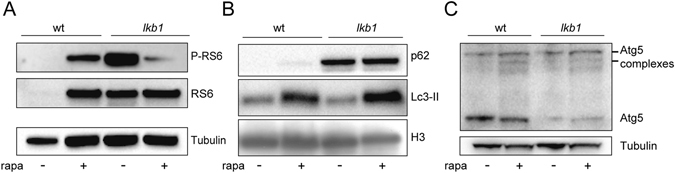
Rapamycin treatment leads to increased Lc3-II accumulation but does not increase Atg5 (complexes) nor restore p62 degradation in *lkb1* mutants. (**A**) Representative Western blot analysis of Ribosomal protein S6 (RS6), Phospho-RS6 and Tubulin (loading control) in total protein lysates of wt and *lkb1* trunks at 6 dpf that were treated with either 10 μM rapamycin from 24 hpf onwards, or with DMSO (negative control). Increased levels of RS6 and P-RS6 are observed in rapamycin-treated wt samples. Total RS6 levels did not change in *lkb1* samples but P-RS6 decreased upon rapamycin treatment. (**B**) Representative Western blot analysis of p62, Lc3-II, and histone H3 (loading control). Larvae were treated with chloroquine (2.5 μM) for 14 h prior to processing. Rapamycin treatment leads to increased Lc3-II levels in both wt and *lkb1* larvae, while p62 accumulation remained high in rapamycin-treated *lkb1* mutants. (**C**) Representative Western blot analysis of Atg5 and Tubulin (loading control). Rapamycin treatment leads to an increase in the amount of the ~47 KD Atg5-containing complex in wt larvae and to a lesser extent in *lkb1* mutants. Uncropped images of the blots are shown in Supplementary Fig. S9A–C.

We next investigated the mechanism behind the impaired autophagy observed in the *lkb1* mutants. Signaling through mTOR is known to inhibit autophagy, and we and others have previously reported that mTOR activity is high in wt larvae between 2 and 5 dpf and is downregulated at later stages of larval development^37, 47, 48^. This suggests that at the time of yolk depletion, mTOR activity is switched off, enabling the activation of autophagy. It has also been shown in mice that suppression of mTOR activity at birth enables activation of autophagy^49^. We hypothesized that mTOR inactivation was defective in the absence of Lkb1. Therefore, we first assessed the status of mTOR signaling in *lkb1* mutants at the metabolic transition (6 dpf) by analyzing phosphorylation of the mTOR-substrate ribosomal protein S6 (RS6) by Western blot. Total RS6 levels were almost undetectable in wt larvae at this stage, consistent with our previous report^37^. However, both total and phospho- RS6 levels were high in *lkb1* mutants (Fig. 3A), indicating active mTOR signaling, which was inhibited by rapamycin treatment. In comparison, in rapamycin-treated 6 dpf wt larvae, we observed increased phospho-RS6 expression (Fig. 3A). This could be explained by a known developmental delay caused by chronic mTOR inhibition during development^50^. Consistent with this, rapamycin-treated wt larvae retained significant amounts of yolk at 7 dpf, demonstrating a delay in larval development (Supplementary Fig. S4C).

To determine whether mTOR signaling mediates the inhibition of autophagy seen in the *lkb1* mutants at 6 dpf, we examined whether rapamycin treatment could restore autophagy in these mutants. We treated wt and *lkb1* embryos with rapamycin from 1 dpf onwards. We have previously reported that rapamycin-treated *lkb1* larvae survive until 9 dpf, but still have a considerable amount of yolk, demonstrating a developmental delay^37^. Rapamycin-treatment resulted in elevated Lc3-II levels in both wt and *lkb1* larvae, at 6 dpf (Fig. 3B), indicating that autophagy in the *lkb1* mutants is inhibited, at least in part, by mTOR signaling. The same was also observed when blocking autophagosome-lysosome fusion by chloroquine, which prevents degradation of autophagosome-associated Lc3, allowing monitoring of the autophagic flux^51^ (Supplementary Fig. S4A,B). However, rapamycin-treatment led to only slight upregulation of Atg5 and complexed Atg5 in *lkb1* mutants (Fig. 3C), and was not sufficient to decrease the marked p62 accumulation (Fig. 3B). These results suggest that while mTOR inhibition can at least partially restore autophagy in *lkb1* mutant larvae, it cannot entirely alleviate the observed phenotype.

**Figure 4:**
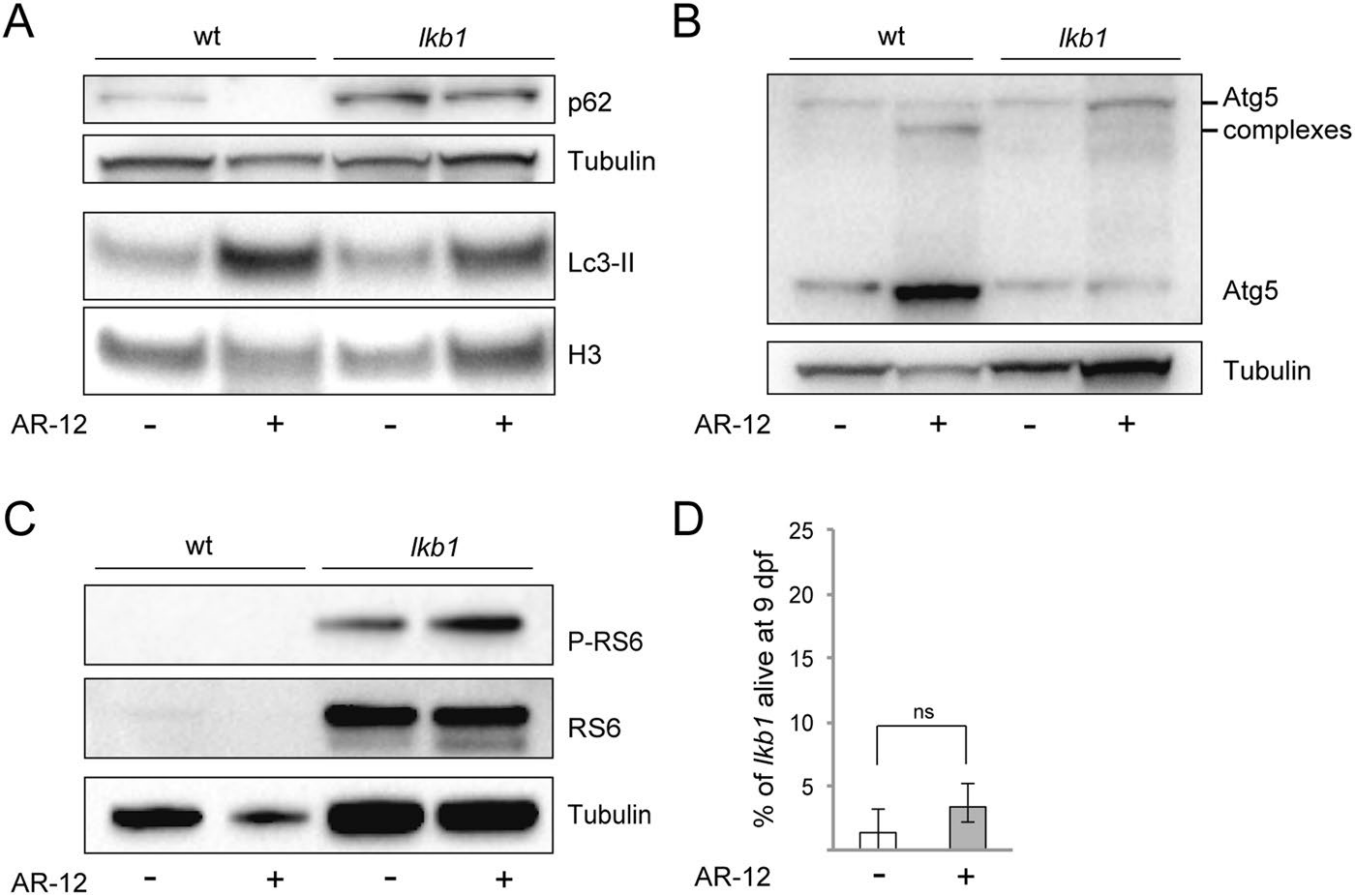
The PI3K-inhibitor AR-12 does not rescue the autophagy defect in *lkb1* mutants and does not prolong their survival. (**A**) Representative Western blot analysis of Lc3-II, p62 and Histone H3 and Tubulin (loading controls) in total protein lysates of wt and *lkb1* trunks at 6 dpf. Larvae were treated with 1 μM AR-12 or DMSO (negative control) from 1 dpf, and with 2.5 μM chloroquine for 14 h prior to processing. AR-12 treatment leads to upregulation of Lc3-II levels in wt larvae but not in *lkb1* mutants. p62 levels remain high in AR-12-treated *lkb1* larvae. (**B**) AR-12 treatment leads to strong increase in the amounts of Atg5 and complexed Atg5 in wt larvae but not in *lkb1* mutants. Uncropped images of the blots are shown in Supplementary Fig. S10A,B. (**C**) Representative Western blot analysis of Ribosomal protein S6 (RS6), Phospho-RS6 and Tubulin (loading control). Protein levels of total RS6 and of P-RS6 were not affected by AR-12 treatment in *lkb1* mutants. Uncropped images of the blots are shown in Supplementary Fig. S9A–C. (**D**) Graph depicting survival percentage of *lkb1* larvae alive at 9 dpf. Embryos were treated with 1 μM AR-12 or DMSO from 1 dpf, collected at 9 dpf, and genotyped for the *lkb1* gene. Data represent the means ± standard errors of the means (SEM) and are pooled from three independent experiments P value > 0.05, ns: not statistically significant.

We next analyzed the pro-survival PI3K pathway, which in response to external stimuli (growth factors, insulin) also suppresses autophagy, acting upstream of mTOR signaling^52^. To this end, we used the small molecule AR-12, an inhibitor of phosphoinositide-dependent kinase (PDK)-1, a component of the PI3K pathway^53^, which has been shown to activate autophagy in zebrafish^54^. Treatment of wt and *lkb1* siblings with AR-12 from 1 dpf onwards, led to accumulation of Lc3-II protein levels in wt but not in *lkb1* larvae at 6 dpf (Fig. 4A). Interestingly, AR-12 treatment resulted in upregulation of Atg5 expression and formation of Atg5/12 complexes in wt larvae indicating autophagy induction, but no changes in Atg5 expression were observed in *lkb1* larvae (Fig. 4B). Furthermore, while p62 protein expression was diminished in wt larvae upon AR-12-mediated activation of autophagy, accumulation of p62 remained unchanged in AR-12-treated *lkb1* larvae (Fig. 4A). This indicates that inhibition of the PI3K pathway fails to induce autophagy in mutant larvae. We next assessed phosphorylation of the mTOR-substrate RS6 upon AR-12 treatment in wt and *lkb1* larvae at 6 dpf. RS6 and phosphorylated RS6 (P-RS6) were not detectable in wt larvae at 6 dpf (Fig. 4C), consistent with downregulation of mTOR activity at later developmental stages (here and refs 37, 47 and 48). AR-12 treatment did not affect the high protein levels of RS6 or P-RS6 seen in the *lkb1* mutants, indicating that they are unsusceptible to PI3K pathway inhibition. In line with the lack of autophagy induction, AR-12 treatment did not enhance *lkb1* survival, as no statistically significant differences were observed in the percentage of AR-12-treated *lkb1* larvae alive at 9 dpf compared to DMSO-treated controls (Fig. 4D).

**Figure 5:**
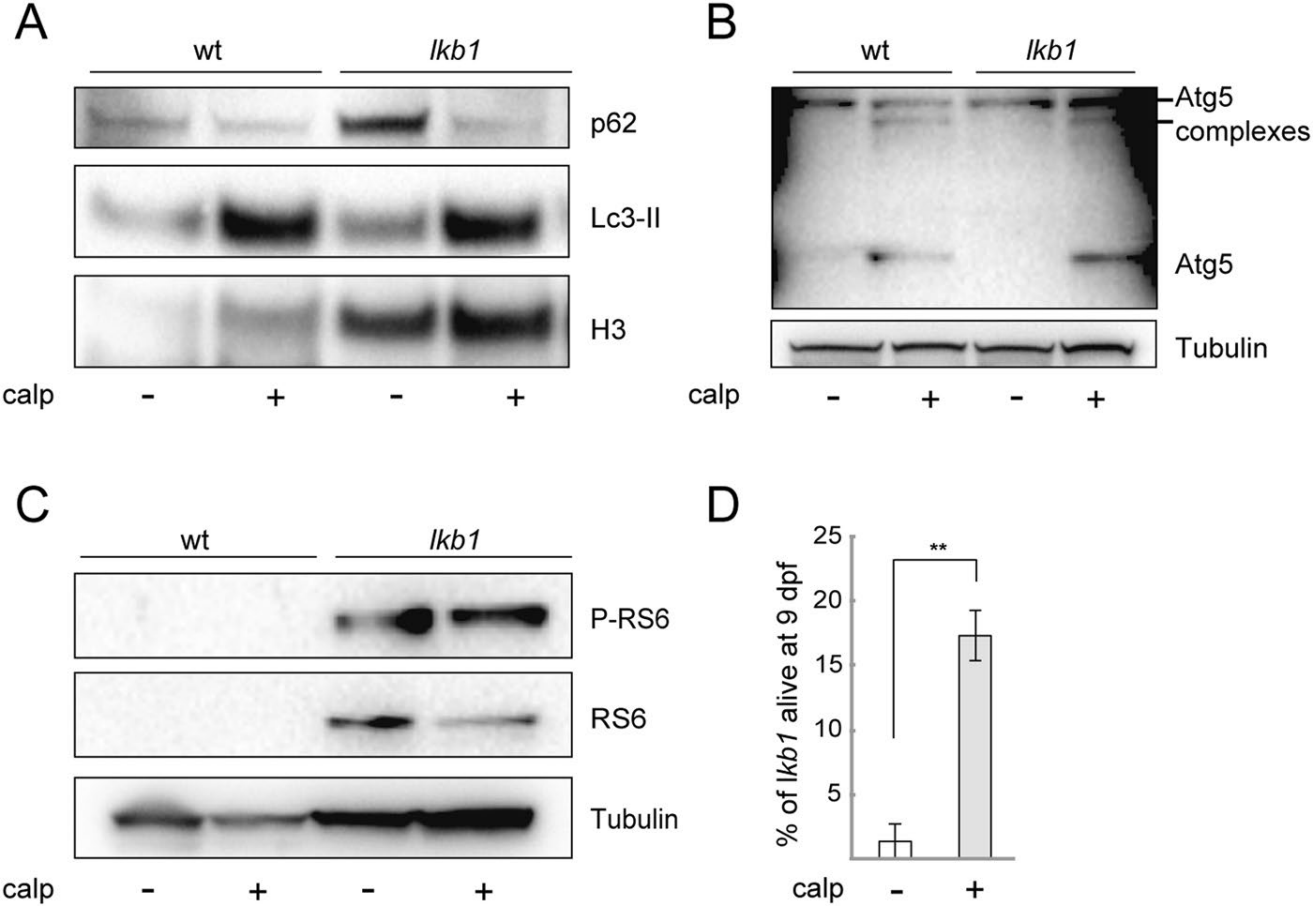
Calpeptin treatment induces autophagy and prolongs survival of *lkb1* larvae. (**A**) Representative Western blot analysis of Lc3-II, p62 and Histone H3 (loading control) in total protein lysates of wt and *lkb1* trunks at 6 dpf. The embryos were treated with 50 μM calpeptin or DMSO (negative control) from 1 dpf onwards, and with 2.5 μM chloroquine for 14 h prior to lysing. Calpeptin treatment leads to upregulation of Lc3-II levels in both wt and *lkb1* larvae. Induction of autophagy by calpeptin also leads to robust downregulation of p62 accumulation in *lkb1* larvae. (**B**) Representative Western blot analysis of Atg5 and Tubulin (loading control). Calpeptin treatment leads stark increase in the amounts of Atg5 and of complexed Atg5 in both wt and *lkb1* larvae (**C**) Representative Western blot analysis of Ribosomal protein S6 (RS6), Phospho-RS6 and Tubulin (loading control). Calpeptin treatment does not affect RS6 or P-RS6 levels in wt or *lkb1* mutants. Uncropped images of the blots are shown in Supplementary Fig. S11A–C. (**D**) Graph depicting survival percentage of *lkb1* mutants alive at 9 dpf. Embryos were treated with 50 μM calpeptin or DMSO from 1 dpf, collected at 9 dpf, and genotyped for the *lkb1* gene. 17,5% out of a total 25% (70% of calpeptin-treated *lkb1* larvae) are alive at 9 dpf. Only 1% of DMSO-treated *lkb1* larvae are alive at 9 dpf. Data depicted in (**D**) represent the means ± standard errors of the means (SEM) and are pooled from three independent experiments (n = 100/experiment). **P value < 0.05.

Activation of autophagy by an mTOR-independent pathway can be achieved using calpeptin, which inhibits the autophagy inhibitors calpain proteases^55^. Calpeptin treatment of *lkb1* and wt embryos from 1 dpf onwards, in the presence or absence of chloroquine, enhanced Lc3-II levels in both wt and *lkb1* larvae at 6 dpf (Fig. 5A and Supplementary Fig. S5) without any effects on development or yolk absorption. Calpeptin treatment also resulted in upregulation of the amounts of Atg5 and complexed-Atg5 in both wt and *lkb1* mutants (Fig. 5B). In contrast to rapamycin treatment, treatment with calpeptin restored p62 degradation in *lkb1* mutants (Fig. 5A). Calpeptin treatment had no effect on RS6 phosphorylation in *lkb1* mutants (Fig. 5C), consistent with calpeptin being an mTOR-independent autophagy activator^55^. Moreover, calpeptin-mediated activation of autophagy prolonged survival in 70% of the treated *lkb1* larvae. Specifically, 17.5% of calpeptin-treated *lkb1* larvae survived until 9 dpf, whereas only 1% of vehicle-treated *lkb1* larvae were alive at this point (Fig. 5D). Therefore, we conclude that induction of mTOR-independent autophagy results in a more complete restoration of the *lkb1* mutant phenotypes compared to that obtained upon inhibition of mTOR signaling.

### Accumulation of p62 is an important regulator of autophagy in *lkb1* mutants

**Figure 6:**
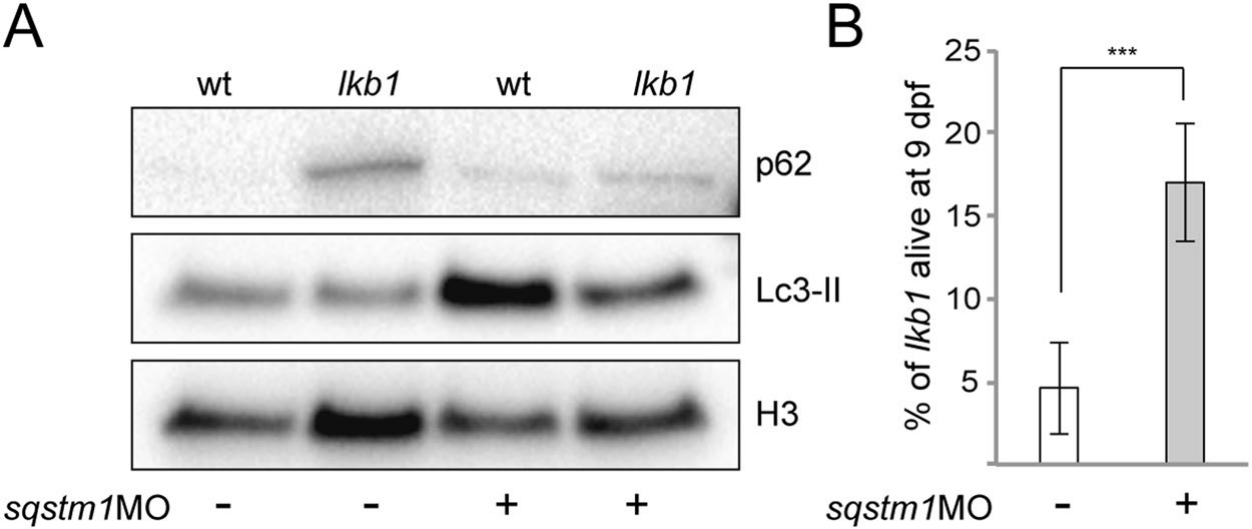
p62 knock-down extends *lkb1* mutants’ survival. (**A**) Representative Western blot analysis of p62, Lc3-II, and histone H3 (loading control) in total protein lysates of wt and *lkb1* trunks at 6 dpf that were either injected with an *sqstm1*MO at the one-cell stage or controls. The larvae were treated with 2.5 μM chloroquine for 14 h prior to processing. p62 knock-down leads to increased Lc3-II levels in both wt and *lkb1* larvae. p62 expression in *lkb1* larvae is reduced upon p62 knock-down. Uncropped images of the blots are shown in Supplementary Fig. S12. (**B**) Graph depicting survival percentage of *lkb1* larvae alive at 9 dpf. Embryos were injected with 0,5 mM *sqstm1*MO at the one cell stage, collected at 9 dpf, and genotyped for the *lkb1* gene. Data represent the means ± standard errors of the means (SEM) and are pooled from three independent experiments. ***P value ≤ 0.0001.

Aberrant p62 accumulation appeared as a hallmark of impaired autophagy in *lkb1* mutants, and strongly correlated with survival. While p62 is primarily thought of as a receptor delivering cargo proteins to autophagosomes for degradation, it has also been implicated in enhancing mTOR activity^56^, thereby regulating autophagy as well. Loss of p62 function led to increased autophagy in mammalian cells and in *C. elegans* ^56^. We thus set out to determine whether reducing p62 levels in larvae would affect autophagy and survival. To this end, we injected a *sqstm1/p62* MO, targeting splicing of *sqstm1*/*p62* mRNA^54^, into 1–2-cell stage embryos. RT-PCR confirmed that the *sqstm1/p62* MO blocked *sqstm1/p62* mRNA splicing until at least 5 dpf (Supplementary Fig. S6). Western blot analysis of 6 dpf larvae showed decreased p62 expression compared to un-injected controls in both wt and *lkb1* lysates (Fig. 6A). This was coupled with increased Lc3-II protein levels, suggestive of autophagy induction. Knockdown of p62 significantly prolonged *lkb1* survival up to 9 dpf: Approximately 70% of *sqstm1/p62* MO-injected *lkb1* mutants survived to 9 dpf, whereas less than 5% of un-injected *lkb1* larvae were alive at this time-point (Fig. 6B). Thus, depleting p62 is sufficient to activate impaired autophagy in *lkb1* mutants and extend survival.

## Discussion

Organisms adapt their metabolism in response to nutrient limitation to restore energy homeostasis and ensure survival. Here, we identify a novel link between metabolic adaptation during development and induction and maintenance of autophagy, mediated by the tumor suppressor Lkb1. Specifically, we use metabolically compromised Lkb1-deficient zebrafish larvae to show that Lkb1 is crucial in the induction of autophagy in response to the metabolic challenge accompanying depletion of the maternal nutrient supply. Our data therefore reveal an essential function for Lkb1 in controlling starvation-induced autophagy at the organismal level in vertebrates.

Overall autophagy levels in *lkb1* mutants are lower compared to those of wt siblings: while expression of autophagy–related proteins is progressively upregulated following yolk depletion in wt larvae, induction of autophagy in *lkb1* mutants is strongly attenuated. Importantly, we demonstrate that genetic and chemical manipulation of autophagy levels significantly impacts *lkb1* larvae survival: inducing autophagy by mTOR-dependent and –independent mechanisms prolongs survival, and suppressing autophagy by Atg5 depletion leads to premature death selectively of the mutants. The increased susceptibility of *lkb1* larvae to Atg5 depletion during development occurred even while the yolk is not yet consumed, suggesting that even though the larvae do not show a morphological phenotype at this embryonic stage, the loss of Lkb1 appears to sensitize them to additional stress. This stress may be specifically autophagy inhibition, or related to alternative mechanisms, as autophagy-independent functions have been reported for several of the autophagy-related genes^57, 58^, including Atg5^59^.

Various mechanisms, including mTOR and PI3K signaling, as well as calpains, are known to regulate autophagy^19^, and likely interact at multiple levels. Indeed, our results, together with published work, indicate that all these influence the energy-sensing defect we observe in *lkb1* mutants. We show that activating autophagy by calpeptin, which inhibits the action of the general autophagy inhibitors calpains^55^, led to robust upregulation of Atg5 expression and restored degradation of p62 in *lkb1* mutants. Thus, calpeptin fully rescued the autophagy defect of the *lkb1* larvae and prolonged their survival. In contrast, while the mTOR-inhibitor rapamycin increased Lc3-II accumulation in *lkb1* larvae, autophagy was not completely restored since p62 still accumulated. This may be due to the high mTOR activity in the mutants that could not be fully blocked by rapamycin treatment under these experimental conditions. In addition, although rapamycin treatment also prolonged *lkb1* survival, we believe this was likely due to a generalized growth delay, evidenced by the presence of a considerable amount of yolk at 7 dpf (this study and refs 37 and 50), rather than due to partial restoration of autophagy. A developmental delay caused by rapamycin is further supported by the persistence of RS6 expression in rapamycin-treated wt larvae at 7 dpf when mTOR would normally be suppressed (mTOR signaling is suppressed in wt larvae upon yolk depletion at 5–6 dpf^37, 47, 48^).

The autophagy receptor and substrate p62 aberrantly accumulates in *lkb1* mutants indicating deficient autophagy. p62 is also a regulator of autophagy, as it participates in a feed-forward loop in which p62 enhances mTOR activity resulting in reduced autophagy, in turn leading to higher p62 levels in mice^60^. Here we also show that depletion of p62 in *lkb1* larvae leads to activation of autophagy and prolonged survival. This implies that as the amount of p62 decreases due to autophagosomal clearance, its effect on mTOR activity is also reduced, and thus autophagy can be maintained. Furthermore, the aberrant accumulation of p62 in *lkb1* larvae may in itself contribute to their premature lethality, as it has been shown that increased levels of p62 in autophagy-deficient mouse livers cause hepatotoxicity (reviewed in ref. 60). Further supporting our hypothesis, in apoptosis-impaired tumor cells with deficient autophagy, p62 accumulation triggers a positive feedback loop for the generation of reactive oxygen species (ROS) leading to enhanced genomic instability and tumorigenesis^61^.

PI3K signaling is a nutrient-sensing pathway that is also implicated in starvation-induced autophagy. Inhibition of the PI3K pathway activated autophagy in wt larvae, but not in *lkb1* mutants, and did not prolong their survival. This is consistent with our previous findings that PI3K signaling is compromised in *lkb1* mutants^37^. We postulate that defective PI3K signaling may contribute to the autophagy defect seen in these mutants. While AMPK is considered a major regulator of metabolism and has an important role in induction of autophagy under energetic stress^23, 44^, it is not overtly activated in wt larvae at 7 dpf^37^; in agreement with these data, studies in mice have also reported that 24 hours of fasting did not lead to significant AMPK activation^62, 63^. Thus, the autophagy defect we describe in *lkb1* mutants is unlikely to be solely attributable to impaired AMPK signaling, and deregulation of additional pathways, such as PI3K signaling and AMPK/mTOR-independent pathways may also be involved. Hence, nutrient-sensing pathways (like the PI3K pathway) and energy-sensing pathways (like the AMPK pathway) are likely in close cross-talk with each other, not only through their convergence on mTOR signaling but also through different, mTOR-independent mechanisms.

Together, our data indicate that Lkb1 plays an important role in the regulation of autophagy at the whole-organism level, and confirm that autophagy is critical for survival during the metabolic transition in development. Since defects in autophagy are implicated in a plethora of diseases, a better understanding of the upstream regulatory pathways could provide new insights into their pathophysiology.

## Materials and Methods

### Zebrafish strains and Screening Methods

Zebrafish were handled in compliance with the local animal welfare regulations and were maintained according to standard protocols (zfin.org). Their culture was approved by the local animal welfare committee (DEC) of the University of Leiden and all protocols adhered to the international guidelines specified by the EU Animal Protection Directive 2010/63/EU. Genotype analysis for *lkb1* mutants embryos was performed as previously described^37^.

### Longitudinal analysis of survival of *lkb1* mutants

Larvae obtained from single matings of heterozygous *lkb1* adults were analyzed over time. 48–95 larvae were genotyped on 6, 7, 8, 9, 10 and 11 dpf to assess the numbers of *lkb1* mutants alive.

### Western Blot analysis

Approximately 20 larvae/sample were lysed (3 μl per larva) in cold lysis buffer (50 mM Hepes, pH 7.6, 50 mM KCl, 50 mM NaF, 5 mM NaPPi, 1 mM EGTA, 1 mM EDTA, 1 mM beta-Glycerophosphate, 1 mM DTT, 1 mM Vanadate, 1% NP40) containing phosphatase and proteinase inhibitors. Lysates were pestled for 5 min, sonicated for 30 seconds at 30 seconds intervals for 5 min and centrifuged at 13.000 rpm for 15 min at 4 °C to pellet nuclei and cell debris. Protein lysates were boiled for 10 min and BCA assay was performed to measure protein concentration. Samples containing 12–30 μg of protein were heated at 95 °C for 5 min with 4× Bolt LDS sample buffer (Thermofisher, #B0007), supplemented with 5% beta-mercaptoethanol, and loaded onto a 12% Bis-Tris plus gel (Thermofisher, #NW00122). The protein marker used was Precision Plus Protein^TM^ Dual Color Standards, #1610374 (BioRad). Proteins were transferred onto a nitrocellulose membrane (Thermofisher, #88018) using a wet transfer system (Bio-Rad) according to manufacturer’s instructions. Subsequent blocking and antibody incubation were performed in 5% skimmed milk powder (#115363, Merck Millipore) in PBS containing 0.1% Tween-20. For the anti-p62 antibody, blocking was performed in 10% milk powder and antibody incubation in 1% milk powder in PBS containing 0.1% Tween-20. Antibodies used were: rabbit anti-LC3B (1:1000, Abcam, #ab51520), rabbit anti-p62 (1:1000, MBL, #PM045), rabbit anti-BECN1 (1:500, Santa Cruz, #sc-11427), rabbit anti-H3 (1:5000, Santa Cruz, #sc-10809), mouse anti-beta-actin (1:5000, Sigma, #A5441), mouse anti-Tubulin (1:500, Sigma, #T9026), rabbit anti-Atg5 (1:500, Novus, #NB110-53818). Secondary antibodies used were goat Anti-Mouse IgG (H + L)-HRP (1:10.000, BioRad, #1721011) and Goat Anti-Rabbit IgG (H + L)-HRP Conjugate (1:10.000, BioRad, #17210191). Membranes were developed using ECL (BioRad, #1705060), followed by chemiluminescence detection with a gel doc system (BioRad).

### Immunohistochemistry and Immunofluorescence

For transverse sections, larvae were fixed in 40% ethanol, 5% acetic acid and 10% formalin for 3 h at room temperature followed by three washes in 70% ethanol before being dehydrated following serial washes in Histoclear and reducing ethanol concentrations. Larvae were then sectioned at 5 μm intervals using a Reichert-Jung 2050 microtome (Leica). Sections were deparaffinized and hydrated following by 20 min of antigen retrieval in sodium citrate buffer pH 6.0 at 100 °C. Sections were blocked in 5% BSA in PBS − 0.1% Tween-20 for 1 h at room temperature and incubated overnight with sheep anti-p62 (1:200, Abcam, #ab31545) and rabbit anti-BECN1 (1:150, Santa Cruz, #sc-11427). Endogenous peroxidase activity was blocked in 0.3% H_2_O_2_ for 20 min at room temperature followed by incubation with rabbit anti-sheep antibody (1:800, Abcam, #ab6747) for 1 h at room temperature. Sections were incubated with 0.1 M imidazole prior to detection with 3,3′-diaminobenzidine (DAB) substrate and counterstaining with hematoxylin.

For immunofluorescence, larvae were fixed in 4% PFA overnight at 4 °C, embedded vertically in a 0.5% gelatin/30% albumin mixture and sectioned at 120 μm intervals using a VT1000S vibratome (Leica). Sections were transferred to the wells of a 24-well plate containing PBD (PBS + 0.1% Tween-20 and 0.5% Triton-X-100), which was then replaced with blocking solution (PBD + 1% BSA) for 1 h at RT. Sections were incubated with rabbit anti-LC3B (1:1000, Abcam, #ab51520) in blocking solution overnight at 4 °C. Sections were washed three times for 15 min in PBS-0.1% Tween-20 and incubated with secondary anti-rabbit 488 green fluorescent antibody (1:100, Thermofisher, #A11008) for 2 h at room temperature. Sections were then washed three times for 15 min in PBS-0.1% Tween-20 prior to be incubated with phalloidin-Alexa 588 (1:25, Thermofisher, #A12380) and DAPI (1:200, Thermofisher, #62248) for 30 min at room temperature in the dark and rinsed three times for 5 min with dH_2_O. Sections were then imaged using the Zeiss LSM5 Exciter confocal laser-scanning microscope.

### Equipment and settings

For immunohistochemistry, the sections were imaged on an upright compound Nikon Eclipse E800 microscope. The images were captured using a Nikon Digital Sight camera unit, equipped with a DS-Fi1 digital camera head and a DS-L2 camera controller. Pixel dimensions of the acquired images were W2584 X H1936 pixels, at 150 pixels/inch.

The magnification used was either 40×/0,75 magnification for anti-p62 staining (Fig. 1) or 100×/1,4 magnification for anti-Becn1 staining (Supplementary Fig. S2).

The images were processed using Photoshop CS6 software. The original images were scaled-down constraining proportions, and cropped to the area of interest. Adjustment of Image Levels was applied on whole images. Assembly of the composite figures and labeling was done on Illustrator CC2015.

Confocal images were obtained in a sequential manner using a Zeiss LSM5 Exciter Confocal Laser Scanning Microscope equipped with Argon (458, 488, 514 nm), and 405, 450 and 635 diode excitation lasers and a 40× water immersion objective (C-APOCHROMAT 40×/1.2 Water). Emission ranges were set at 420–480, 505–550 and 560–615 nm in separate channels to prevent bleeding. Images were obtained using the Leica application X software (Leica, Wetzlar, Germany) and post-acquisition data analysis was performed using ImageJ software.

### Morpholino injections

Translation-blocking morpholino (MO) directed against *atg5* (CATCCTTGTCATCTGCCATTATCAT) was obtained from Gene-Tools. The splice-blocking MO against *Sqstm1/p62* (CTTCATCTAGAGACAAAGTTCAGGA) was a kind gift from Prof. AM Meijer. Splice efficiency of *sqstm1* mRNA was tested in RT-PCR using a specific primer-set (Forward primer: 5′ ATTTGCAGCGAAAAGTGCTC 3′; Reverse primer:

5′ AGTGAACGGAAACCCAGGAA 3′). Embryos were injected at the 1–2-cell stage with either 2 ng (*atg5*) or 4 ng (*Sqstm1/p62*) of MO.

### Drug treatments

Wild type or *lkb1* mutant zebrafish embryos were treated from 1 dpf in embryo-medium at 28 °C with either of the following treatments: 50 μM calpeptin (Abcam, #4ab120804), 1 μM AR-12 (Medkoo Biosciences, #200272), or 10 μM rapamycin (Sigma, #R0395). Stock solutions of AR-12, rapamycin and calpeptin were prepared in DMSO and diluted in embryo medium for treatment (final concentration of DMSO, 0.2%). Other treatments were prepared in embryo medium. All treatments were refreshed every 2–3 days, larvae collected at the specified time points and genotyped for the *lkb1* gene. For Western Blotting, embryos were exposed to 2,5 μM chloroquine (Sigma, #C6628) for 14 h prior to lysing.

### Statistics and quantification

Statistical significance was determined using Fisher’s exact test in GraphPad software. Error bars represent the means ± standard errors of the means (SEM) and are pooled from a minimum of two independent experiments. A p-value of <0.05 was used to define statistical significance.

## Electronic supplementary material

Supplementary material
