# Supplementary material for: The tumor suppressor LKB1 regulates starvation-induced autophagy under systemic metabolic stress
Source: Sci Rep. 2017 Aug 4;7:7327. doi: 10.1038/s41598-017-07116-9 (PMC5544676; doi:10.1038/s41598-017-07116-9)
Supplement: Supplementary file 1 — Supplementary material [file 41598_2017_7116_MOESM1_ESM.pdf]

The tumor suppressor LKB1 regulates starvation-induced autophagy under systemic metabolic stress

Laurie A. Mans[1,&], Laia Querol Cano[1,2,&], Jason van Pelt[1], Panagiota Giardoglou[1], Willem-Jan Keune[3] and Anna-Pavlina G. Haramis[1*]

[1] Institute of Biology, Leiden University, Sylviusweg 72, 2333 BE, Leiden, The Netherlands

[2] Present address: Radboud Institute for Molecular Life Sciences - Tumour immunology department, Geert Grooteplein 28, 6525 GA Nijmegen, The Netherlands.

[3] Department of Biochemistry, Netherlands Cancer Institute, Plesmanlaan 121, 1066 CX Amsterdam, The Netherlands.

[&] These authors contributed equally to the manuscript

[*] Corresponding author, email: a.haramis@biology.leidenuniv.nl

Supplementary Figures

Supplementary Figure 1

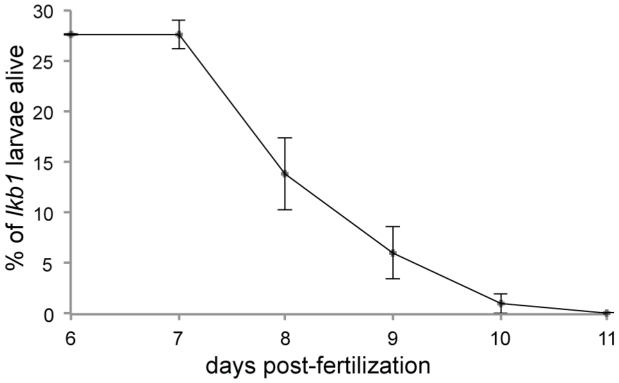

Figure S1. The majority of *lkb1* mutants die from premature starvation at 7-8 dpf. Larvae were collected from pairings of *lkb1* heterozygotes. 100 alive larvae/time-point were collected and genotyped for the *lkb1* mutation at 6, 7, 8, 9 and 10 dpf. The percentage of *lkb1* mutants alive in the total larvae population is plotted on each time-point. Error bars represent the means ± standard errors of the means (SEM) and are pooled from three independent experiments. The graph depicts that *lkb1* mutants start dying at 7 dpf, the majority of *lkb1* mutants die at 8 dpf and very few "escapers" are alive at 9 dpf.

Supplementary Figure 2

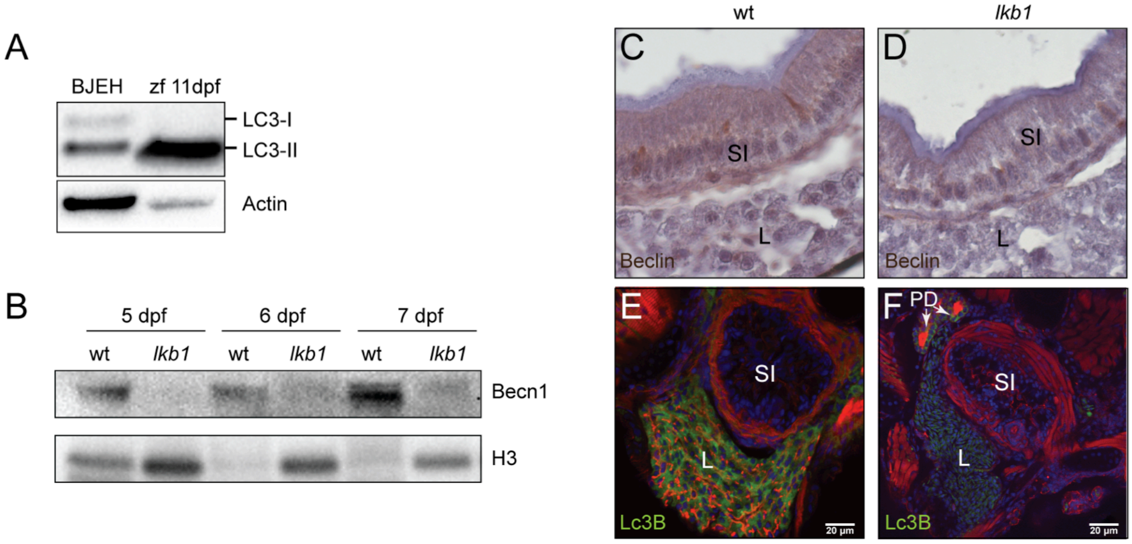

Figure S2. Autophagy markers Lc3B and Beclin are lower in *lkb1* mutants. (A) The LC3B antibody recognizes the cleaved Lc3-II in zebrafish. Western blot analysis using antibodies against LC3B and beta-actin (loading control) on total protein lysates from human BJEH cells that were serum-starved overnight and wt zebrafish larvae at 11 dpf. The LC3B antibody recognizes the uncleaved and cleaved forms of LC3B in the human sample, but predominantly the cleaved Lc3-II in the zebrafish sample. Uncropped images of the blots are presented in Supplementary Fig. S13. (**B**) Western blot analysis of Beclin (Becn1) and Histone H3 (loading control) in total protein lysates of wt and *lkb1* trunks between 5-7 dpf. Larvae were treated with chloroquine (2.5 µM) for 14 h prior to processing. Becn1 levels are lower in the *lkb1* mutants at all time-points. **(C-D)** Immunohistochemical analysis of transverse paraffin sections (5 µm) of intestine of 7 dpf wt and *lkb1* larvae shows very low levels of Becn1 expression in the *lkb1* intestine. Magnification: 100X. **(E-F)** Transverse vibratome sections (150 µm) of liver of 7 dpf wt and *lkb1* mutants stained with anti-LC3B antibody (green), rhodamine-phalloidin to detect F-actin (red), and DAPI to detect nuclei (blue). Lc3B staining in the *lkb1* liver is greatly reduced. PD: pronephric ducts; L: liver; SI: intestine.

Supplementary Figure 3

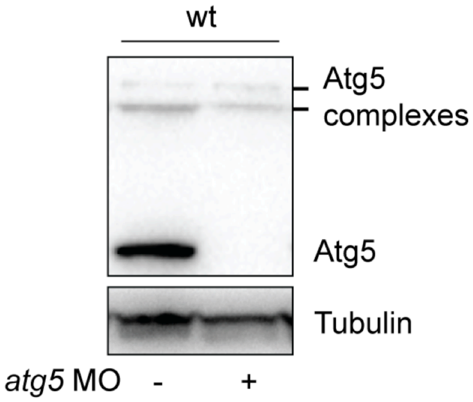

Figure S3. The *atg5*MO efficiently inhibits Atg5 translation. Western blot analysis of wt larvae at 4 dpf, with an antibody against Atg5. Tubulin is used as a loading control. Atg5 expression is almost undetectable in larvae injected with 0,5 mM *atg5*MO at the one-cell stage, showing efficient inhibition of Atg5 translation. Uncropped images of the blots are shown in Supplementary Figure S14.

Supplementary Figure 4

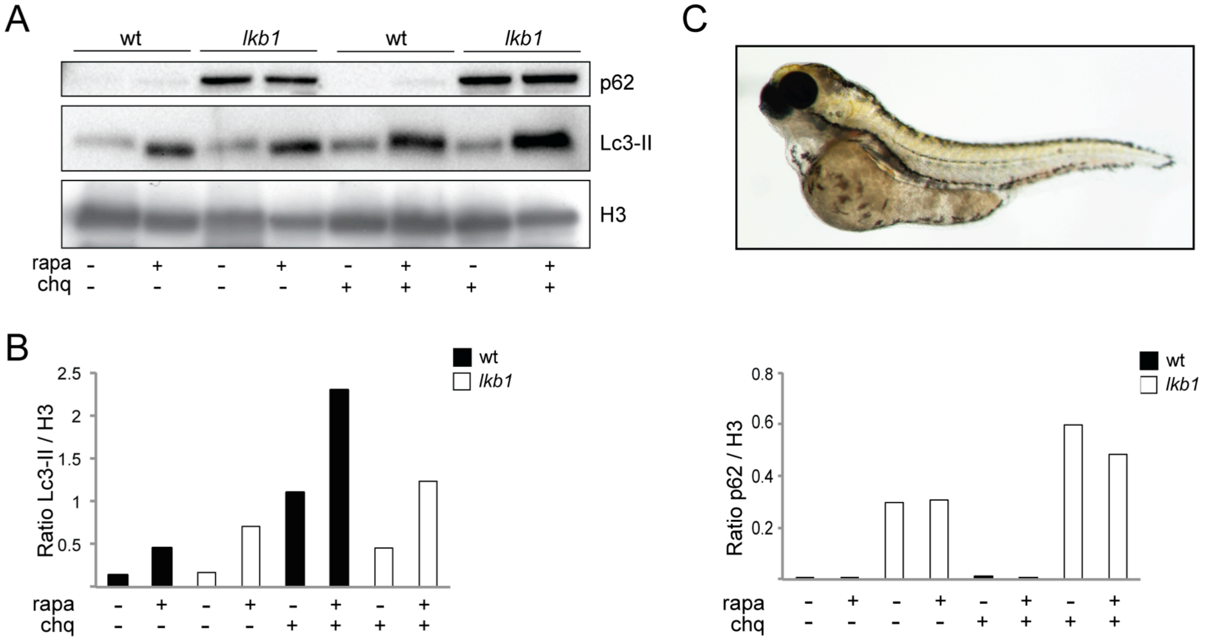

Figure S4. Rapamycin treatment leads to increased Lc3-II accumulation but does not restore p62 degradation in *lkb1* mutants. (A) Western Blot analysis of p62, Lc3-II, and Histone H3 (loading control) in total protein lysates of wt and *lkb1* trunks at 6 dpf that were treated with either 10 µM rapamycin from 1 dpf or DMSO (negative control). To detect the autophagic flux the larvae were treated or not with 2.5 µM chloroquine for 14 h prior to processing. Rapamycin treatment leads to increased Lc3-II levels in both wt and *lkb1* larvae, but p62 levels remained high in the *lkb1* mutants. Uncropped images of the blots are shown in Supplementary Figure S9B. **(B)** Graphical representation of the data shown in (A), depicting the densitometric p62/H3 and Lc3-II/H3 ratios. **(C)** Representative image of a wt larva at 6 dpf that has been treated with 10 µM rapamycin from 1 dpf onwards. Note the high amount of yolk still present indicating developmental delay.

Supplementary Figure 5

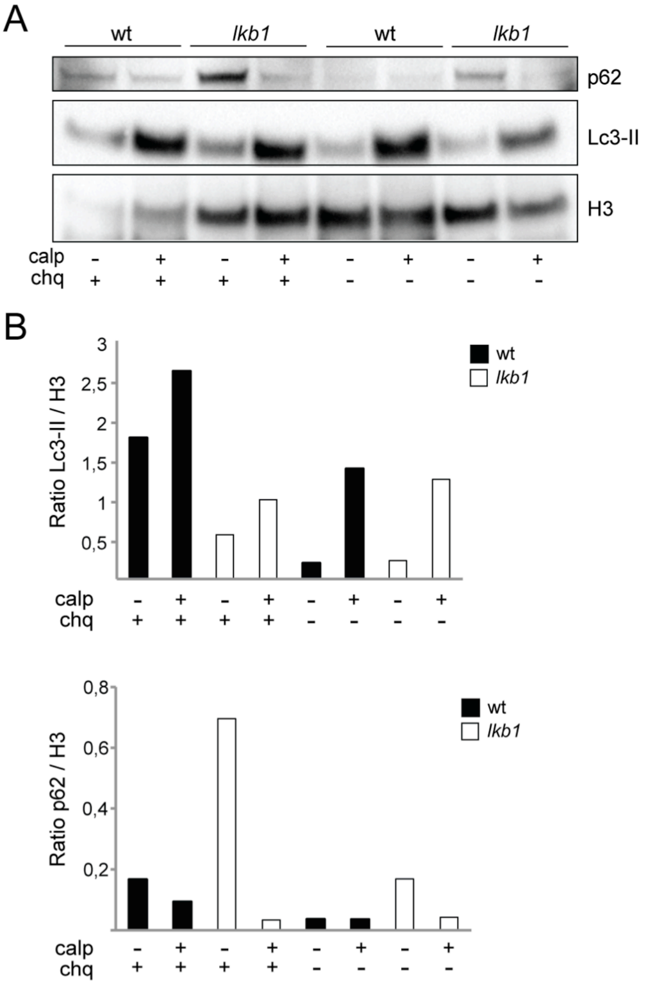

Figure S5. Calpeptin-mediated activation of autophagy restores p62 degradation in *lkb1* mutants. (A) Western blot analysis of Lc3-II, p62 and Histone H3 (loading control) in total protein lysates of wt and *lkb1* trunks at 6 dpf. The embryos were treated with 50 µM calpeptin or DMSO (negative control) from 1 dpf onwards. To detect the autophagic flux, larvae were treated or not with 2.5 µM chloroquine for 14 h prior to lysing. Calpeptin treatment leads to upregulation of Lc3-II levels in both wt and *lkb1* larvae. Induction of autophagy by calpeptin also leads to robust downregulation of p62 accumulation in *lkb1* larvae. Uncropped images of the blots are shown in Supplementary Figure S11A. **(B)** Graphical representation of the data shown in (A), depicting the densitometric p62/H3 and Lc3-II/H3 ratios.

Supplementary Figure 6

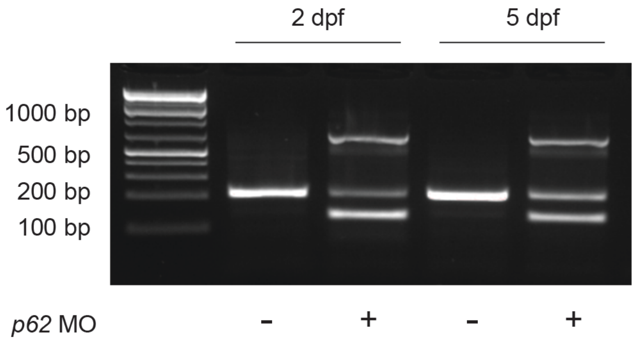

Figure S6. The *sqstm1/p62*MO efficiently blocks splicing of *sqstm1/p62* mRNA. Reverse transcription polymerase chain reaction (RT-PCR) was used to confirm antisense morpholino blocking of intron-exon splicing events in zebrafish *sqstm1/p62* mRNA at 2 and 5 dpf. Upon injection of 0,5 mM *sqstm1/p62* MO (i1e2, targeting splicing between the first intron and the second exon), the 200 bp RT-PCR product is disrupted.

Supplementary Figure 7

A Uncropped images of the blots presented in Figure 1 (Atg5, Tubulin)

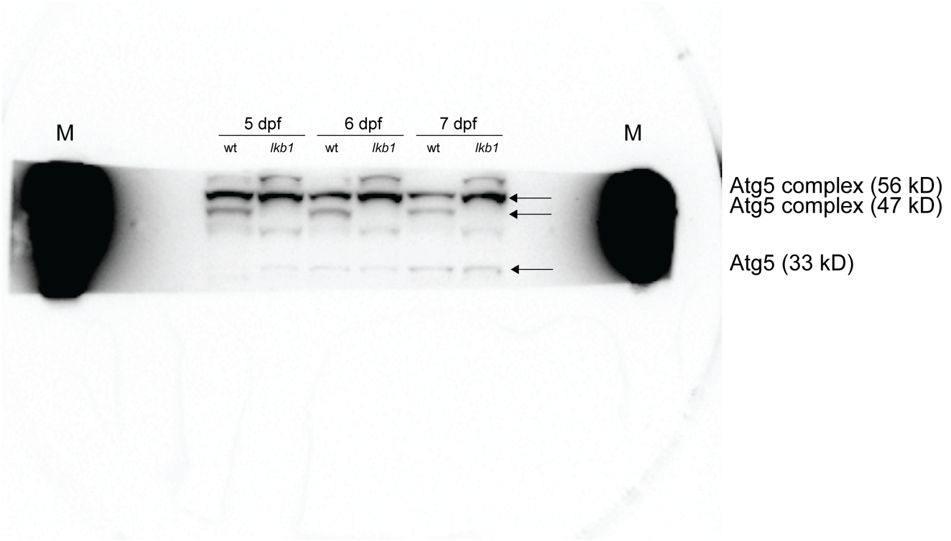

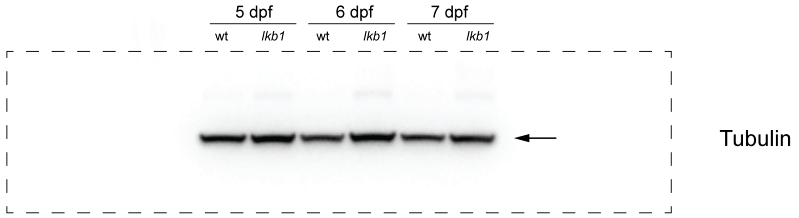

The dashed box indicates the borders of the blot

This tubulin blot was used in the final cropped image

Supplementary Figure 7

B Uncropped images of the blots presented in Figure 1 (p62, Tubulin)

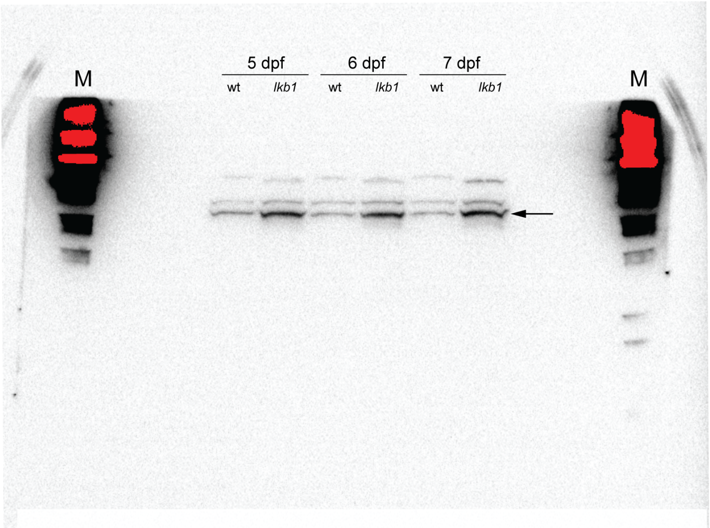

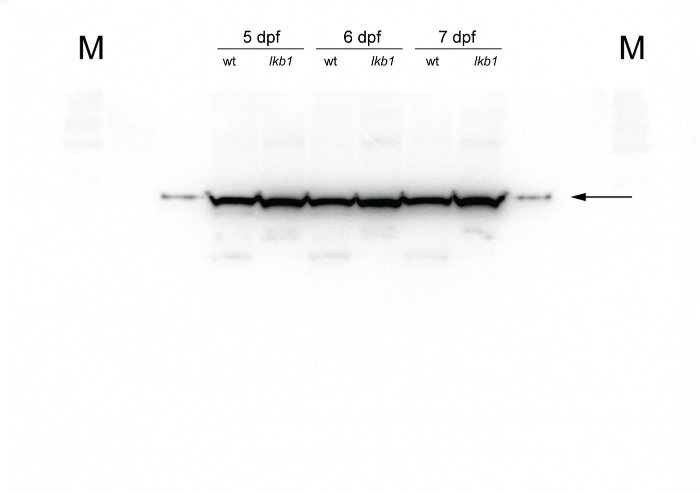

Supplementary Figure 7

C

Uncropped images of the blots presented in Figure 1 (Lc3, Tubulin)

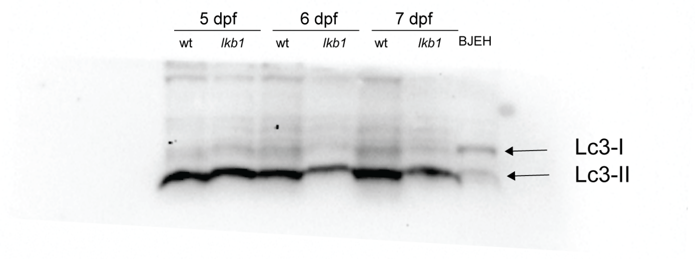

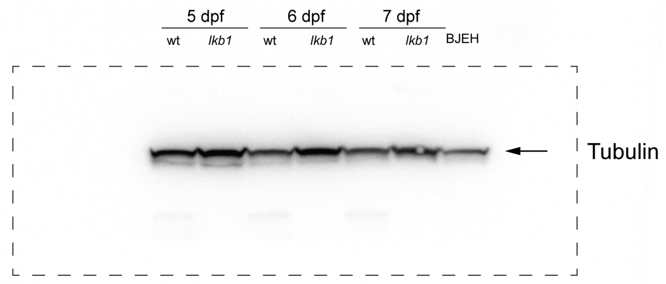

The dashed box indicates the borders of the blot

Supplementary Figure 8

Uncropped images of blots presented in Figure 2

Lc3 II, Actin, upon *atg5*MO-injection

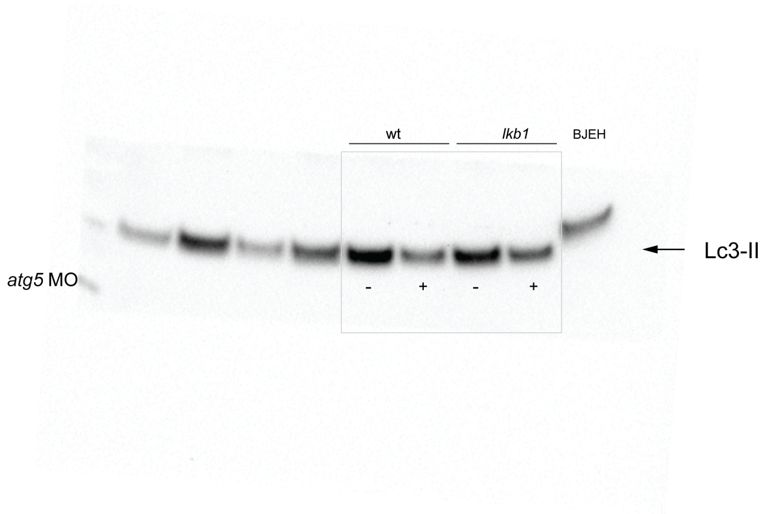

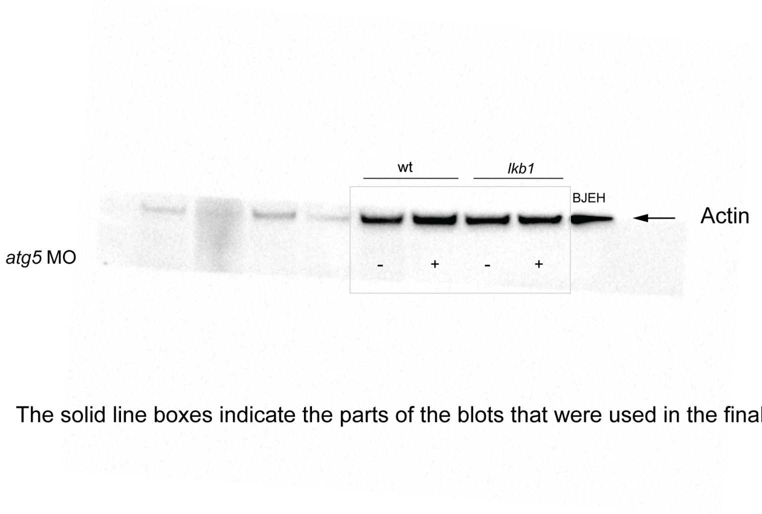

The solid line boxes indicate the parts of the blots that were used in the final cropped image

Supplementary Figure 9

A Uncropped images of blots presented in Figures 3A and Figure 4C

RS6, P-RS6 and Tubulin upon AR-12 and Rapamycin treatments

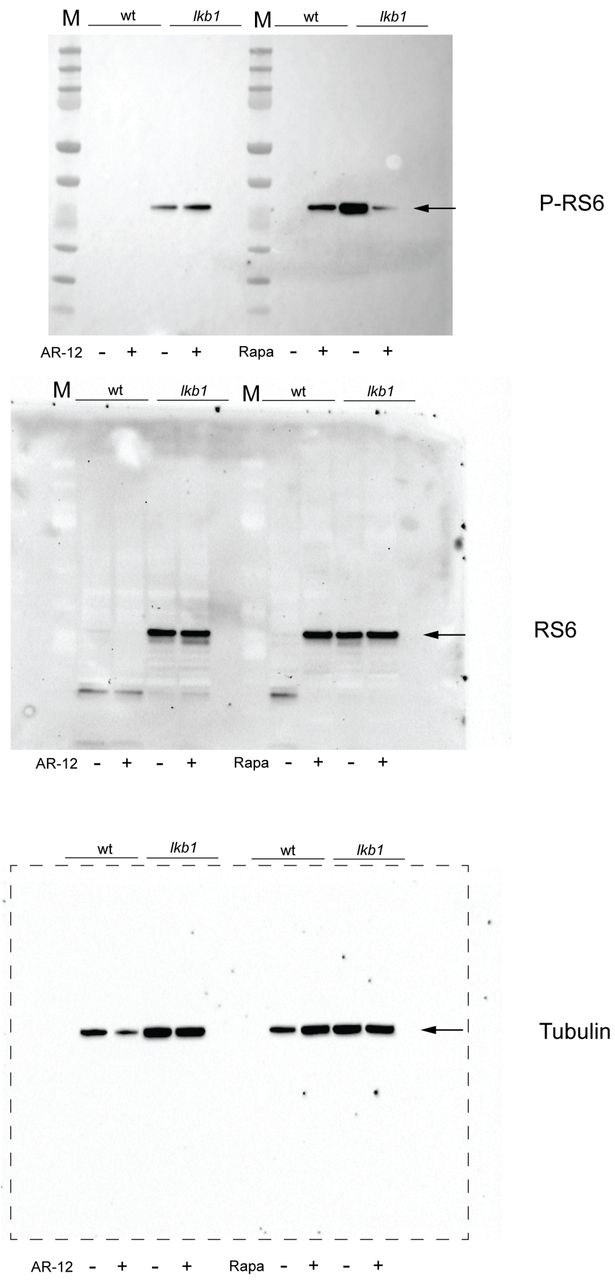

The dashed box indicates the borders of the blot.

Supplementary Figure 9

B

Uncropped images of blots presented in Figure 3B and Supplementary Figure S4A

p62, Lc3 II and H3 upon Rapamycin treatment, +/- chloroquine

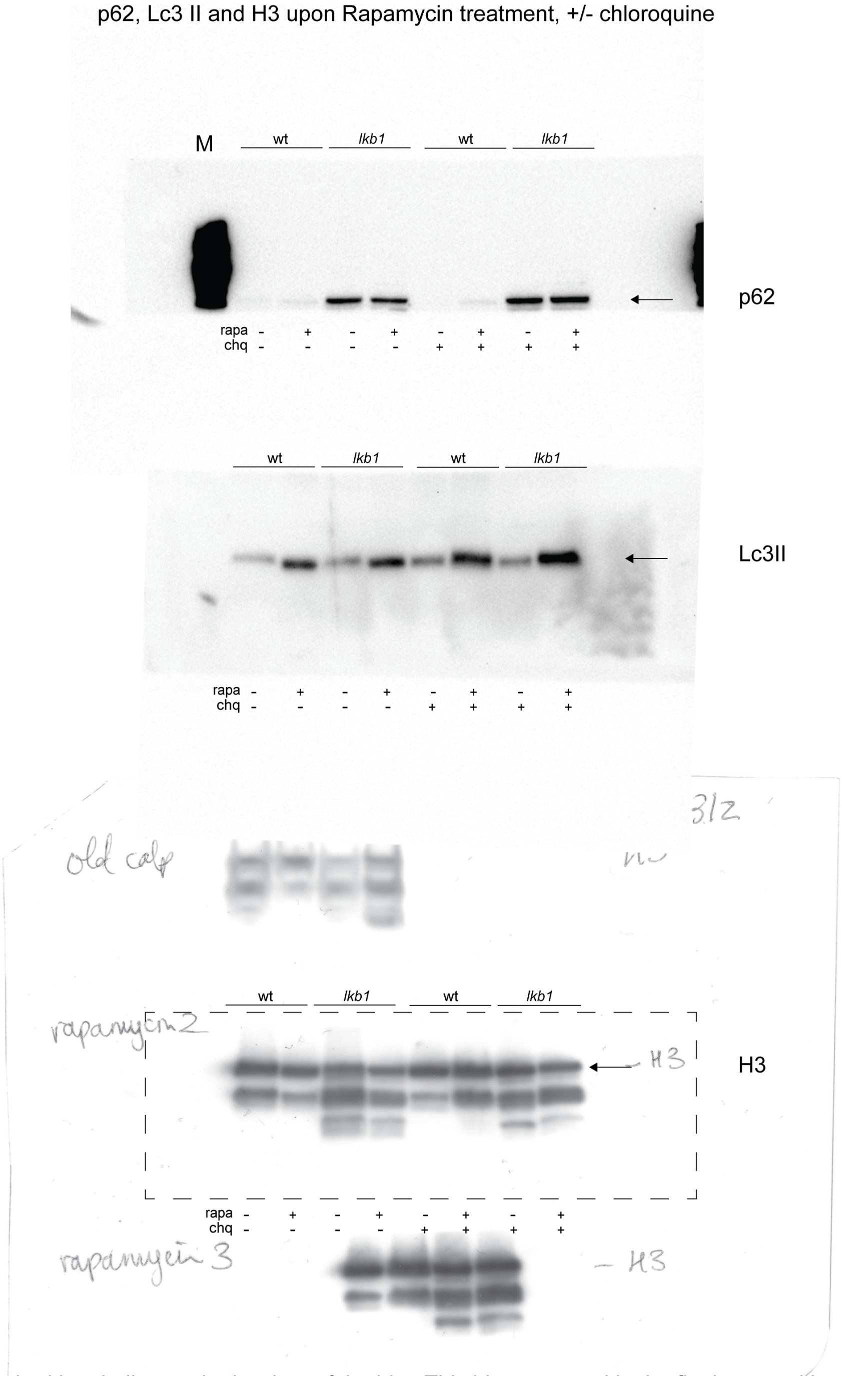

The dashed box indicates the borders of the blot. This blot was used in the final cropped image

Supplementary Figure 9

C

Uncropped images of blots presented in Figures 3C and 4B

Atg5 and Tubulin upon AR-12 and Rapamycin treatments

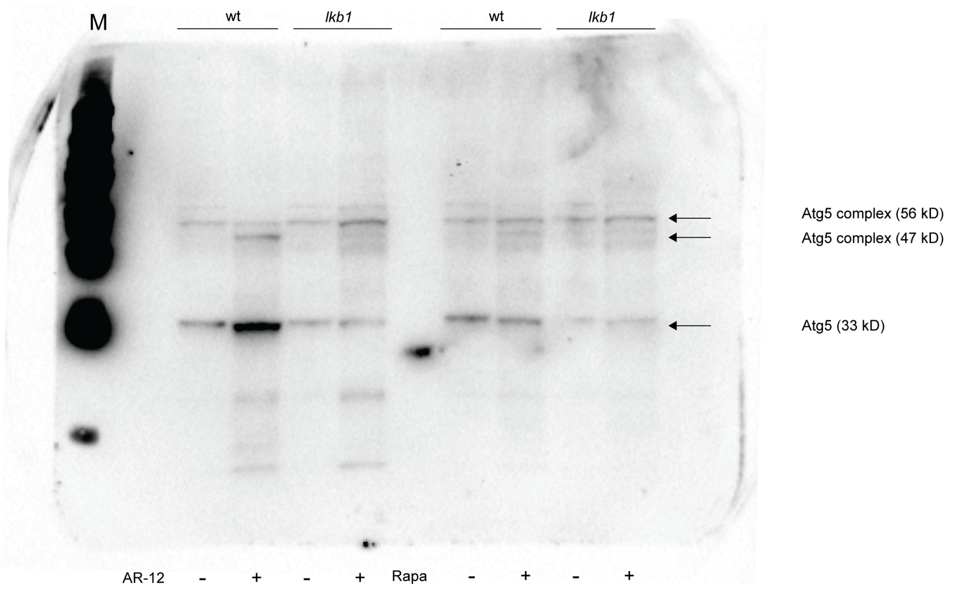

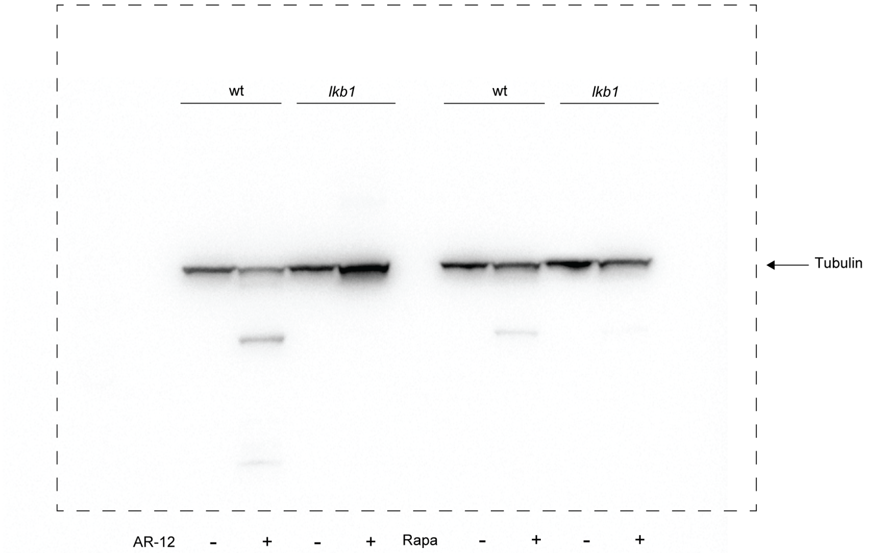

The dashed box indicates the borders of the blot.

Supplementary Figure 10

A Uncropped images of blots presented in Figure 4A

p62 and tubulin upon AR-12 treatment

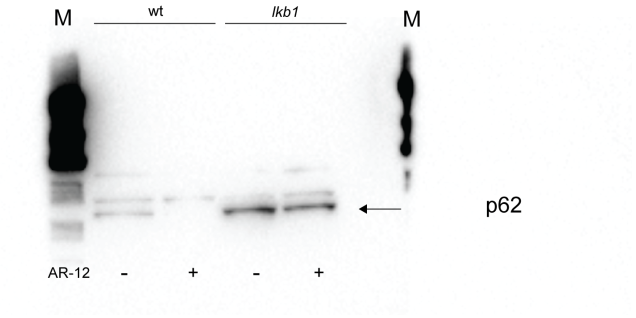

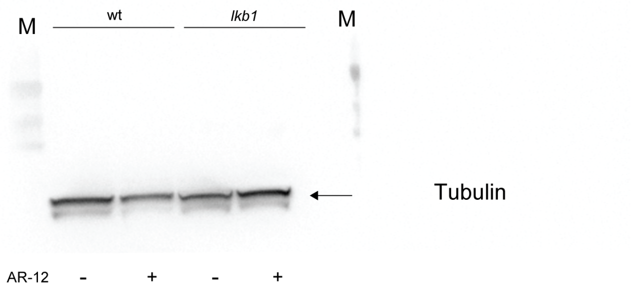

Supplementary Figure 10

B Uncropped images of blots presented in Figure 4A

Lc3 II and H3 upon AR-12 treatment

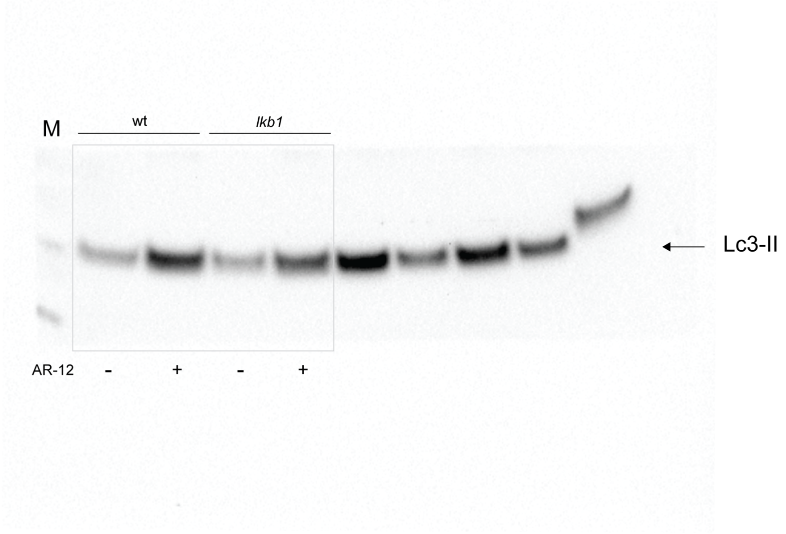

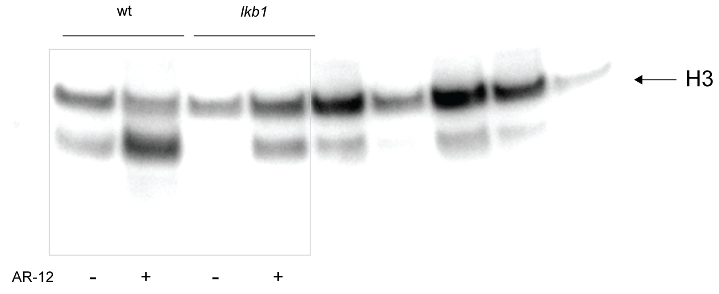

The boxes indicate the parts of the blots that were used in the final cropped image

Supplementary Figure 11

A Uncropped images of blots presented in Figure 5A and Supplementary Figure S5A

p62, Lc3 II, H3 upon calpeptin treatment, +/- chloroquine

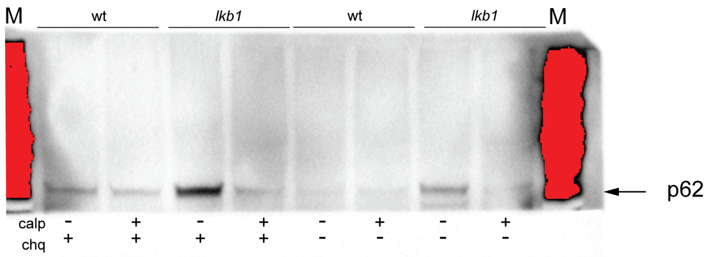

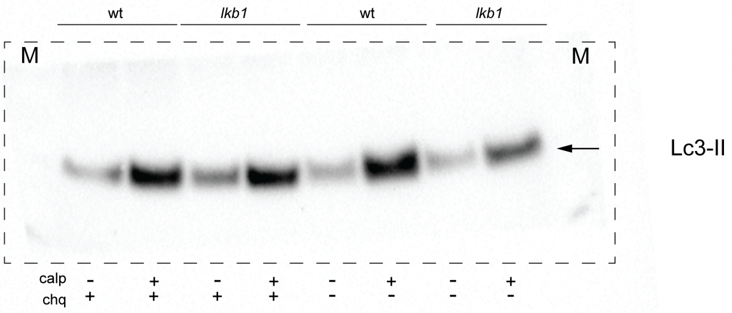

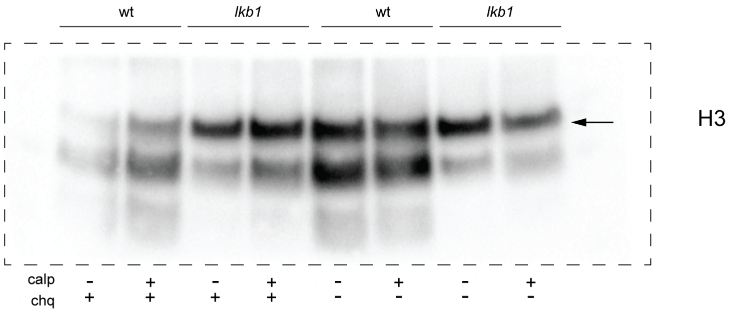

The dashed boxes indicate the borders of the blots

B

Supplementary Figure 11

Uncropped images of blots presented in Figure 5B

Atg5, Tubulin upon calpeptin treatment

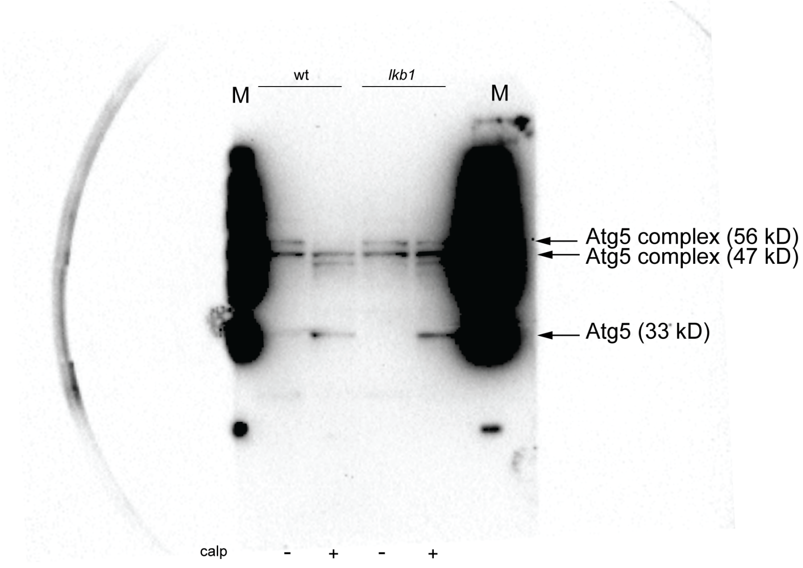

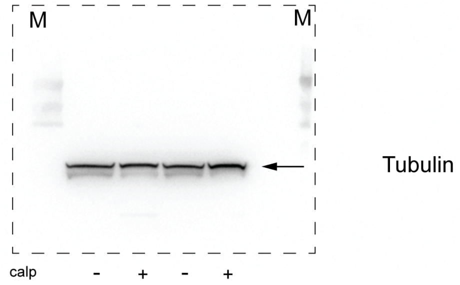

The dashed box indicates the borders of the blot

Supplementary Figure 11

C Uncropped images of blots presented in Figure 5C

RS6, P-RS6 and Tubulin upon calpeptin treatment

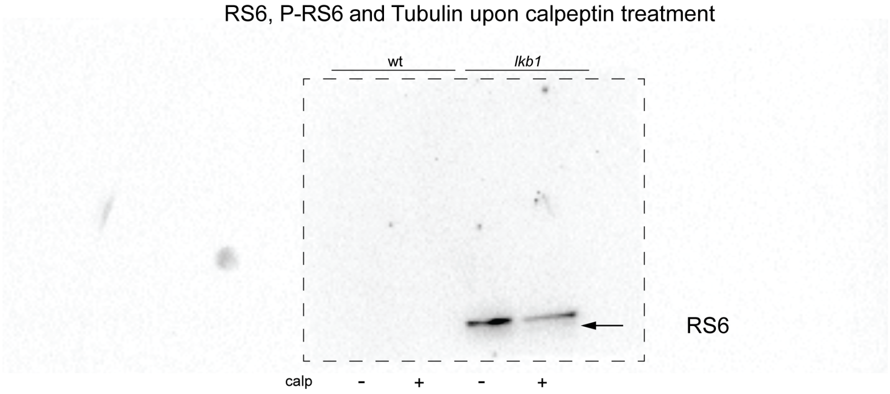

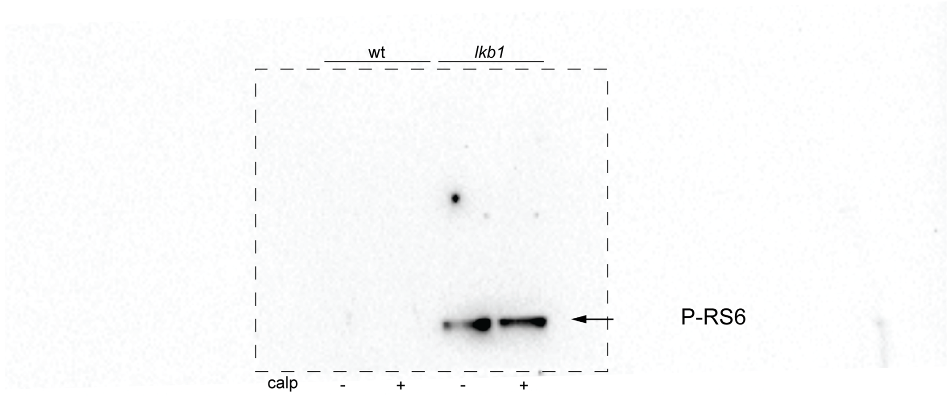

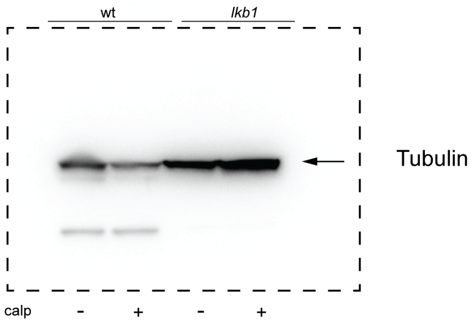

The dashed boxes indicate the borders of the blots

Supplementary Figure 12

Uncropped images of blots presented in Figure 6A

p62, Lc3 II, H3 upon *sqstm1*MO-injection

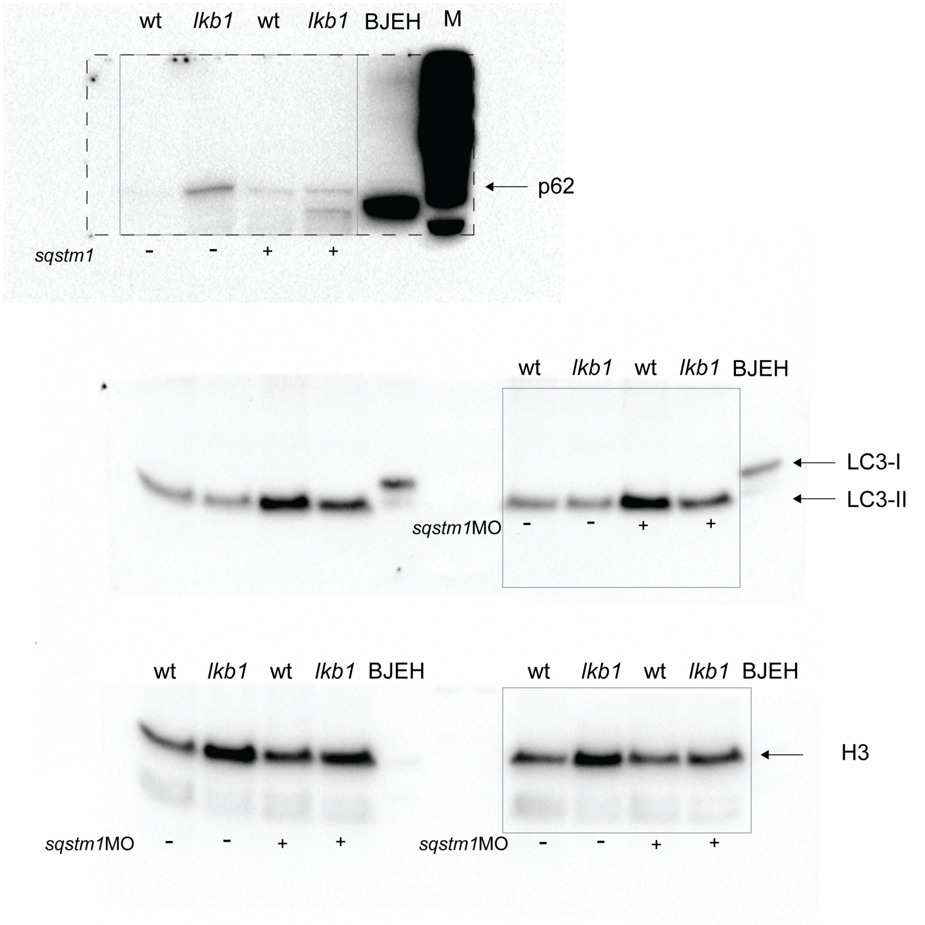

The dashed boxes indicate the borders of the blots

The solid line boxes indicate the parts of the blots that were used in the final cropped image.

Supplementary Figure 13

Uncropped images of the blots presented in Supplementary Figure S2A, B (LC3B Actin, and Becn1 and H3)

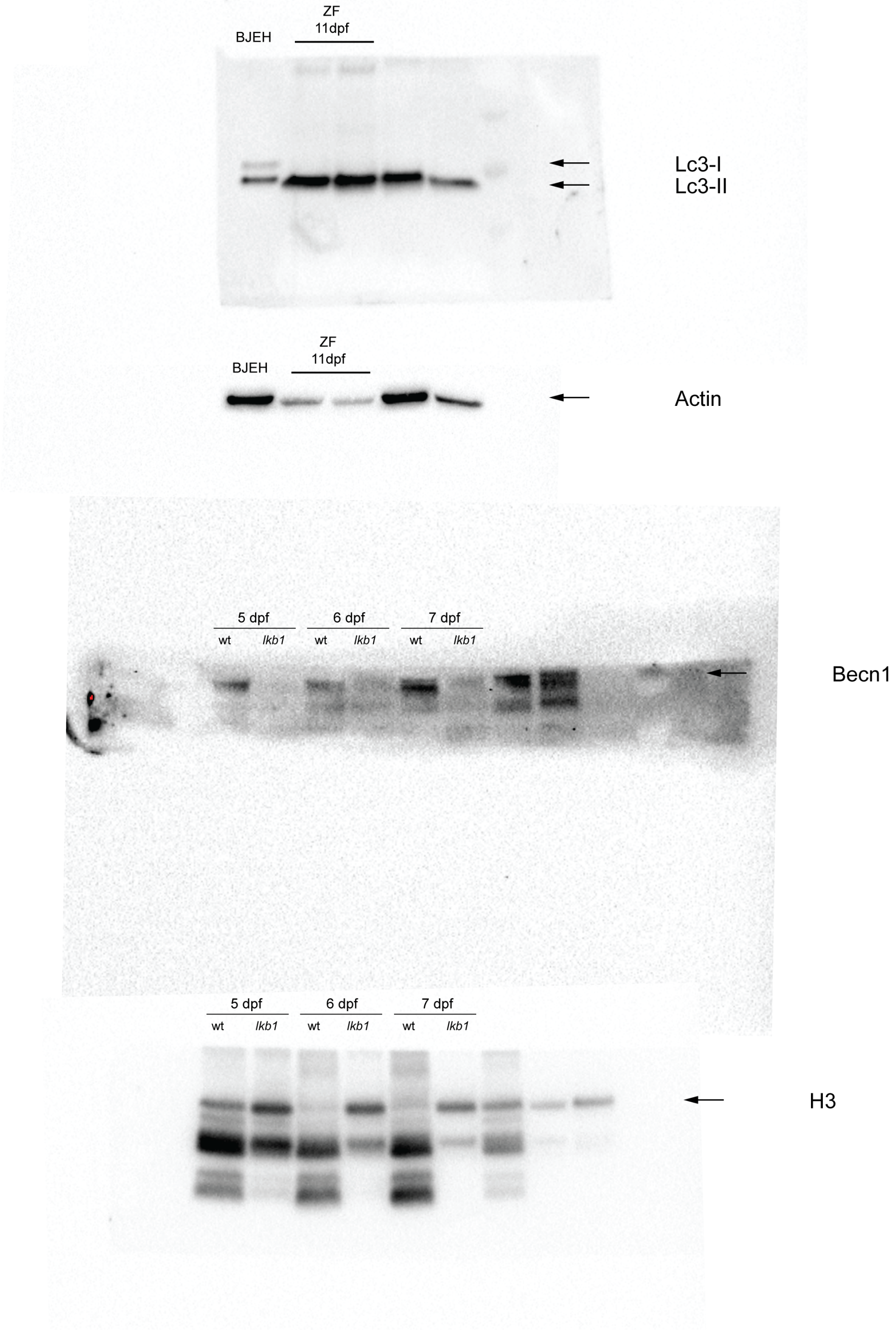

Supplementary Figure 14

Uncropped images of the blots presented in Supplementary Figure S3

Atg5, Tubulin upon *atg5*MO injection

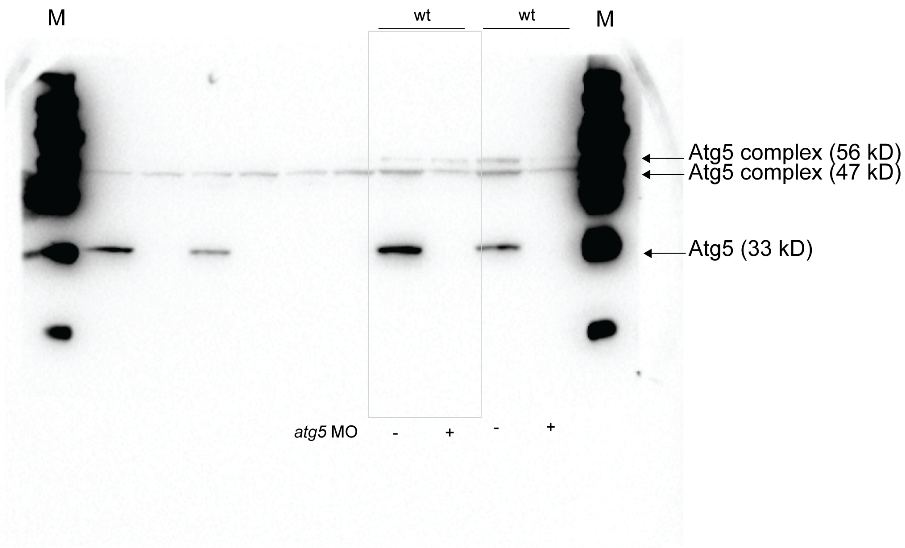

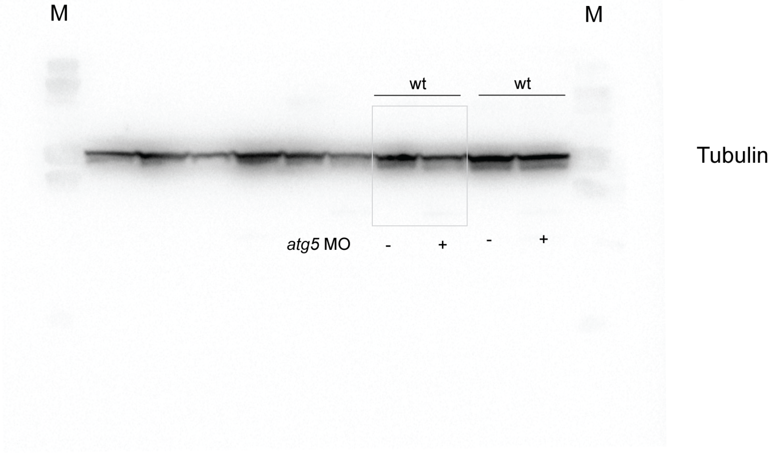

The solid line boxes indicate the parts of the blots that were used in the final cropped image
